# Polysaccharide‐Based Controlled Release Systems for Therapeutics Delivery and Tissue Engineering: From Bench to Bedside

**DOI:** 10.1002/advs.201700513

**Published:** 2018-01-08

**Authors:** Tianxin Miao, Junqing Wang, Yun Zeng, Gang Liu, Xiaoyuan Chen

**Affiliations:** ^1^ State Key Laboratory of Molecular Vaccinology and Molecular Diagnostics and Center for Molecular Imaging and Translational Medicine School of Public Health Xiamen University Xiamen 361102 China; ^2^ School of Chemical & Biomolecular Engineering Georgia Institute of Technology Atlanta GA 30332 USA; ^3^ Collaborative Innovation Center of Guangxi Biological Medicine and the Medical and Scientific Research Center Guangxi Medical University Nanning 530021 China; ^4^ Department of Pharmacology Xiamen Medical College Xiamen 361008 China; ^5^ State Key Laboratory of Cellular Stress Biology Innovation Center for Cell Biology School of Life Sciences Xiamen University Xiamen 361102 China; ^6^ State Key Laboratory of Physical Chemistry of Solid Surfaces and The MOE Key Laboratory of Spectrochemical Analysis & Instrumentation College of Chemistry and Chemical Engineering Xiamen University Xiamen 361005 China; ^7^ Laboratory of Molecular Imaging and Nanomedicine National Institute of Biomedical Imaging and Bioengineering National Institutes of Health Bethesda MD 20892 USA

**Keywords:** clinical translation, drug delivery, polymeric carbohydrate molecules, polysaccharides, tissue engineering

## Abstract

Polysaccharides or polymeric carbohydrate molecules are long chains of monosaccharides that are linked by glycosidic bonds. The naturally based structural materials are widely applied in biomedical applications. This article covers four different types of polysaccharides (i.e., alginate, chitosan, hyaluronic acid, and dextran) and emphasizes their chemical modification, preparation approaches, preclinical studies, and clinical translations. Different cargo fabrication techniques are also presented in the third section. Recent progresses in preclinical applications are then discussed, including tissue engineering and treatment of diseases in both therapeutic and monitoring aspects. Finally, clinical translational studies with ongoing clinical trials are summarized and reviewed. The promise of new development in nanotechnology and polysaccharide chemistry helps clinical translation of polysaccharide‐based drug delivery systems.

## 1 Introduction

The clinical efficacy of low‐molecular‐weight chemotherapeutics and functional biological macromolecules (i.e., proteins and oligonucleotides) is often limited by a number of obstacles, including unfavorable solubility, loss of bioactive structure prior to reaching the disease lesion site, inadequate cellular uptake, short plasma half‐lives due to rapid renal clearance or enzymatic degradation, drug resistance driven by overexpression of the efflux transporter, and unwanted side effects of nonspecific cytotoxic drugs caused by off‐target effect during chemotherapy.^1^ The development of a smart nanoscaled drug delivery system (nanoDDS) has entered the mainstream to not only address these issues but also to aid the advancement of personalized nanomedicine for noninfectious diseases, especially cancer.^2^ With the achievable and tunable size and structure, such nanovehicles can be properly designed to cross the smallest capillary wall while avoiding clearance by a mononuclear phagocyte system (MPS), resulting in a prolonged blood stream duration. Due to the enhanced permeability and retention (EPR) effect,^3^ macromolecules and large nanoparticles can be more effectively trapped in tumor tissues than low‐molecular‐weight molecules and small nanoparticles.^4^ On the other hand, high‐molecular‐weight bioactive molecules (e.g., cytokines and growth factors) have limitations due to their instability in delivery both in vitro and in vivo as well as their immunogenicity and shorter half‐lives. To overcome these limitations, modern drug formulation technologies have facilitated researchers' abilities to create the commonly named “second generation” of protein drugs to overcome the above limitations. Moreover, based on the molecular weight, secondary structure, and availability of surface groups, polymer–protein or fusion protein conjugates have been created. However, protein folding may also be altered through and after the modification process.^6^ Therefore, there is a great need to design delicate DDSs to fulfill the protection of protein therapeutics with enhanced half‐lives and reduced immunogenicity. This strategy can then be used in protein pharmaceutical areas.

For decades, various nanotherapeutics have been developed for use in humans, most of which can be formulated with several main types of DDSs, such as liposomes, micelles, polymeric conjugates, inorganic nanoparticles, and others. Among them, polysaccharides are the most recognized biomaterials that are derived from natural carbohydrate polymers. They are generally regarded as safe (GRAS) and are broadly used in the food industry. Here the term “GRAS” is a general standard defined under sections 201(s) and 409 of the Federal Food, Drug and Cosmetic Act (the Act), meaning any substances that have been generally recognized, among qualified experts and adequately shown to be safe under the conditions of its intended use.^7^ They have also been applied as an excipient for drug formulation by regulatory authorities in different countries.^8^ Basically, polysaccharides are carbohydrates with more than two sugar molecules covalently bonded together by glycosidic linkage. In addition to polysaccharides, there are also monosaccharide and disaccharides within the definition of carbohydrates. They offer a wide range of functional versatility and structural diversity due to their variable molecular weight and abundant reactive groups (i.e., amine, carboxyl, carbonyl, and hydroxyl groups) on the polysaccharide backbone.^9^ Polysaccharides of natural origin commonly exist in various species, including plants (cellulose), animals (chitosan, nature origin obtained from chitin of exoskeletons crustaceans and insects), algae (alginate), and microorganisms (dextran).

**Figure 1:**
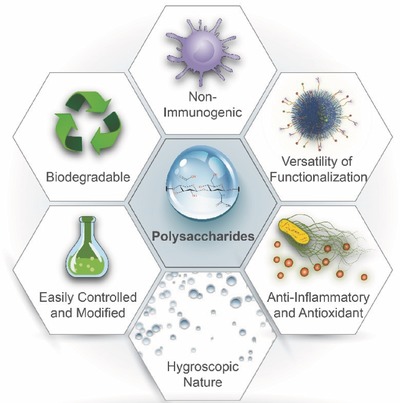
Schematic illustration of unique physiochemical and biological properties of polysaccharides.

Compared to other types of synthetic hydrophobic polymers, polysaccharides hold a large number of hydroxyl groups or other hydrophilic groups, such as carboxyl groups in alginate and amino groups in chitosan, which afford additional aqueous solubility and reinforce bioadhesion and biorecognition characteristics via noncovalent bonding (e.g., electrostatic interactions) between biological tissues and polysaccharides (**Figure** **1**).^10^ For example, chitosan, the only natural positively charged polysaccharide, is capable of attaching to the negatively charged mucosal layers via electrostatic interactions.^11^ Similarly, hyaluronic acid can recognize and bind to the glycoprotein CD44 antigen on cell surfaces.^12^ Moreover, their intrinsic functional moieties can serve as attachment points for chemotherapeutics, imaging probes, and targeting agents using facile chemical modification,^13^, ^14^, ^15^ such as PEGylation and antibody conjugation to provide prolonged circulation time and site‐specific accumulation activities. Furthermore, due to their parallel biochemical properties with human extracellular matrices, polysaccharides are easily recognized and metabolized by the body,^16^ and they have been discovered to be involved in many biological processes including immune recognition and cell signaling,^17^ which are responsible for activation of antimicrobial and anti‐inflammatory responses.^18^ In addition, these biopolymers undergo enzymatic and/or hydrolytic degradation in vivo, leaving innocuous degradation products that can either be reused in biological systems or cleared by the immune system.^19^ Thus, with the aforementioned features, polysaccharides have a promising future as an accessible therapeutic delivery system.

As naturally based biomaterials, polysaccharides have been largely explored for their ability for targeted delivery and control released to improve the therapeutic index of drugs (e.g., chemotherapeutics, antibiotics, proteins, peptides, and nucleic acids) using various routes of administration.^20^, ^21^ The recent advances in polysaccharide‐based nanomaterials have driven apparent trends toward multifunctional and more complex controlled release systems (CRSs), which will take a big step forward in achieving theranostics and regenerative medicine with improved therapeutic efficacy, mechanical properties, and safety profiles.

The aim of this review is to present the state‐of‐the‐art in identification, functionalization, characterization, and application of bioactive polysaccharides originated from natural sources. Various biomedical applications were emphasized including tissue engineering, regenerative medicine, and cancer theranostics. This review will help to explore and investigate novel chemical and biological strategies for functional materials, promoting the clinical translations of polysaccharide‐based materials in biomedical applications.

## 2 Polysaccharides and Chemical Modification for Controlled Release

We have briefly introduced the importance of controlled drug throughout our introduction session. In general, the goal of controlled drug delivery, not only limited to polysaccharide‐based DDSs, are listed as follows. (1) To protect the drug from degradation. This is also being used in the protection of protein‐based biomolecules, such as cytokines and growth factors, which contain sophisticated secondary structure that can be degraded through delivery routes.^22^ (2) To enhance the half‐life of certain drug. A common example is insulin delivery, which requires instant injection after each meal. Dextran–insulin nanoparticles have been created to meet this need.^23^ (3) To maximize the therapeutic effects while reducing the side effects. This is commonly seen in cancer therapy, where chemotherapy/radiotherapy affects patients' body condition severely. We have a chapter in the later session to discuss how researchers have been creative to effective cure cancer with the novel technologies of theranostics.^24^ (4) To take full advantages of existing drug in comparison with identifying a new drug molecular or potential intracellular pharmaceutical target. The research and economic burden to identify a novel drug molecule or to discover a novel signal pathway for drug target is huge. Therefore, researchers revisit some of existing drug molecules, which also have a comprehensive safety/therapeutic profiles as being in market already, and utilize novel drug delivery techniques to make them perform better or for some other type of disease, saving money on both the research and clinical trial stages.^25^

There are many different principles as researchers' guidelines when designing novel DDSs. These principles are not limited to polysaccharide‐based DDSs, but being extensively studied and utilized throughout the design. These drug release mechanisms lead and trigger the design of versatile polysaccharide‐based DDSs with the help of various chemical modification as approaches. (1) The mesh size of the materials could control the diffusion and release of drug molecules.^26^ When the porous structure of the diameter is larger than the drug over three times (γ_mesh_ > 3 × γ_drug_), diffusion is the dominant factor for drug release. Strokes–Einstein equation is usually used to determine the diffusivity (*D*), which depends on the size (radius) of the drug molecule (γ_drug_) and the viscosity of the solution (η) (*R* is the gas constant and *T* is the absolute temperature)^27^

(1) $$D\;=\;\frac{RT}{6\pi \eta \gamma_{\mathrm{drug}}}$$

When the mesh size has similar radius with drug molecule (γ_mesh_  ≈  γ_drug_), drug diffusion will be stalled by steric hindrance.^26^ Therefore, the approach to control the porous size becomes important in designing DDSs. Researchers have controlled the size of porous structure by adjusting the concentrations of polysaccharides or the cross‐linkers. (2) The particle degradation could control the release of drug molecules. Such design of DDSs usually contain degradable cross‐linker.^26^ One common example is 3,3′‐dithiobis(sulfosuccinimidyl propionate), which could be cleaved with reducing agents, such as glutathione (GSH) to achieve intracellular redox drug delivery.^28^ Enzymatic degradation sequence could also be used in design novel degradable cross‐linker. Phelps et al. reported of a protease degradable peptide cross‐linker GCRDVPMSMRGGDRCG, which could be cleaved by matrix metallopeptidase and able to deliver growth factor in vivo.^29^ (3) The material swelling behavior could control the release of drug molecules.^26^ Polysaccharide‐based hydrogel particles will swell to absorb water and the size of the porous structure will be increased, releasing encapsulated drug. Various factors will contribute to the degree of swelling behavior, such as pH,^30^ temperature,^31^ ionic strength,^32^ electric fields,^33^ light,^33^ and glucose,^34^ which have been extensively studied in the field of drug delivery. (4) The mechanical deformation could also induce drug release from the matrix. This strategy is usually designed in cooperation with hydrogel system. By applying mechanical force, hydrogel matrix will deform, leaving enlarged mesh size and triggering convective flow within the network.^35^ Such impulsive change usually create a pulsatile release profile in certain disease scenario, such as insulin delivery after each meal.^26^, ^36^

Based on the chemical composition, structure, solubility, and derivative sources, there are many possible approaches to classify polysaccharides. Considering the chemical composition, we can divide polysaccharides into two categories: (1) homopolysaccharides or homoglycans, which contain a single type of monosaccharides, such as chitin and chitosan, starch, and cellulose; and (2) heteropolysaccharides or heteroglycans, which consist of multiple types of monosaccharides, such as alginate, glycosaminoglycan, hyaluronic acid, and pectin. Other strategy to categorize polysaccharides includes the electronic charge for each specific polysaccharide molecules. For example, chitosan has usually positive charge whereas alginate has negative charge in general. For this chapter, we discuss five different types of polysaccharides based on their origins: chitosan from shellfish,^36^ alginate from algae,^37^ hyaluronic acid from various mammals (human, pig, beef, etc.) and bacteria,^38^ dextran from bacteria,^39^ and cyclodextrin, which are synthetic substances obtained from enzymatic degradation of starch.^40^ We chose these types of polysaccharides as they have been extensively studied and applied in the field of CRS. Molecules with same side groups share similar chemical modifications. In addition, comparable preparation approaches could also be applied to form DDSs. Being as traditional polysaccharide‐based biomaterials, the industrial processing techniques are mature, providing good resources for researchers and clinicians to practice. All the following materials have been going or already gone for clinical trials for various applications. These polysaccharides are good representatives to demonstrate the great research potential and broad applications of polysaccharide materials: from bench to bedside. There are also other types of polysaccharides, which have also shown variety of modification and fabrication potential and biomedical applications, such as carrageenan,^41^ pullulan,^42^ pectin,^43^ cellulose,^44^ starch,^45^ and some new polysaccharides from marine or bacterial origin.^46^ Interested readers are referred to the reviews that we have listed above for more details.

### 2.1 Chitosan‐Based CRS

**Figure 2:**
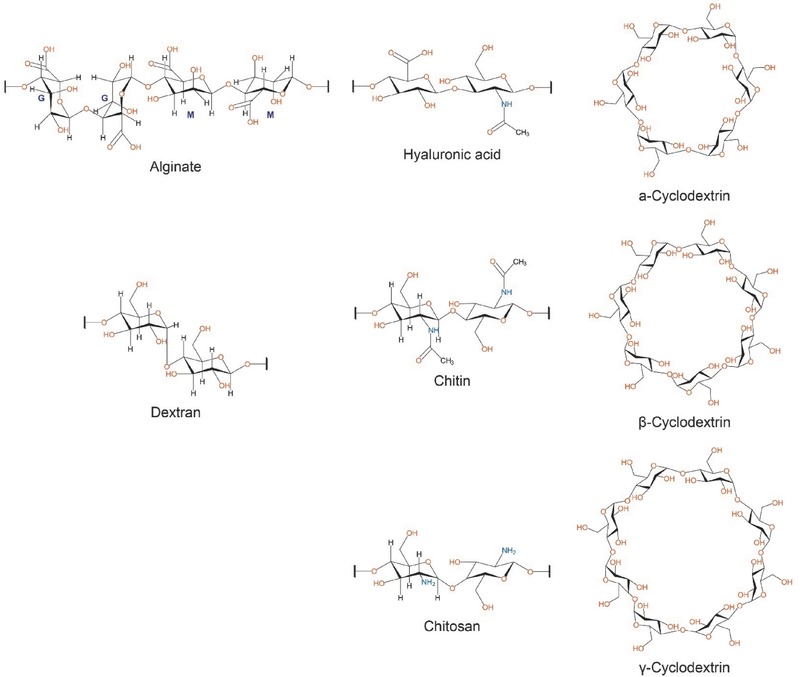
Structures of repeating units of some of the polysaccharides discussed in this review. Branching is not shown for dextran. The structure of alginate and hyaluronic acid are shown the two linkage types rather than a formal repeating unite. The chitin and chitosan structures shown represent extremes of a continuum of structures.

Second to cellulose, chitin is the second most abundant natural amino polysaccharide throughout the world, which is the typical component of shellfish exoskeletons and fungal cell walls. It is a linear cationic polymer of *N*‐acetyl‐d‐glucosamine (2‐acetylamino‐2‐deoxy‐d‐glucose) units that are joined by β‐1,4 linkages.^47^ Chitosan is produced by the deacetylation of chitin, which consists of β‐(1,4)‐linked deacetylated units of d‐glucosamine and *N*‐acetyl‐d‐glucosamine^47^, ^48^ (**Figure** **2**). Chitosan has been studied and used within the pharmaceutical area for almost three decades. As is widely recognized, chitosan‐based nanoplatform is one of the most promising DDSs due to its positive attributes of superior biocompatibility, notable biodegradability (metabolized by lysozyme), low toxicity, and positively charged characteristics based on its primary amino groups (this property enables electrostatic interaction with negatively charged macromolecules, nucleic acids, proteins, mucosal surfaces, etc.).^48^, ^49^ In spite of these advantages, the aqueous solubility of chitosan is relatively poor at neutral pH in some cases.^50^ However, molecular weight and residual acetyl groups in the chitosan may also play important roles in the solubility of chitosan. To overcome the solubility issue, acidic solution (pH <6.5) has been used while introducing additional water solubilizing groups. Reducing the molecular weight and elevating the degree of deacetylation can also facilitate additional solubilization, but this can spontaneously affect the physicochemical properties of chitosan.^51^

Owing to the pH‐induced solubility of chitosan modified drug conjugates, Park et al. developed a pH sensitive glycol chitosan‐based drug conjugate for photodynamic therapy.^52^ This DDS consisted of three functional moieties that grafted on amine groups along the chitosan chain. The photosensitive drug Chlorin e6 and polyethylene glycol (PEG) residues were crosslinked with glycol chitosan through dicyclohexyl carbodiimide (DCC)/*N*‐hydroxysulfosuccinimide (NHS)‐mediated amidation. 3‐Diethylaminopropyl isothiocyanate (DEAP) was then grafted onto the chitosan backbone via thiourea bond formation. DEAP was exploited as an endogenous stimulus for pH triggered drug release in acidic tumor tissue that lead to conformational changes of nanoparticles from a coiled (at pH 7.4) into an uncoiled structure (at pH 6.8). Moreover, the protonation of DEAP residues generates the additional singlet oxygen to provide higher phototoxicity for cancer cells. In another study, a facile and controlled graft polymerization of *N*‐(2‐hydroxyethyl)prop‐2‐enamide and chitosan was achieved by using γ‐ray irradiation of bis(R,R′‐dimethyl‐R″‐acetic acid) trithiocarbonate.^53^ The anticancer drug chromone‐3‐carboxaldehyde was then grafted on the amino groups of chitosan via Schiff‐base bond formation, which was a cleavable covalent bond that undergo hydrolysis at low pH conditions. Specifically, this amphiphilic copolymer conjugate could self‐assemble into micelle nanoparticles in a water solution.

In addition to pH‐sensitive DDSs, Hu et al. described a selective redox‐responsive chitosan‐based glycolipid‐like micelles, which was able to control the drug release rate by GSH concentration in tumor cells.^54^ The polymer was prepared via a two‐step synthesis. First, disulfide linker (bis‐2‐carboxyethyl disulfide) was conjugated with stearylamine through amide bond formation by applying DCC/4‐dimethylaminopyridine (DMAP). Second, the carboxyl‐terminated intermediate product was conjugated to amino groups on chitosan through the 1‐ ethyl‐3‐(3‐dimethylaminopropyl)carbodiimide hydrochloride (EDC a water soluble carbodiimide)/NHS‐mediated coupling reaction. The low pH reaction environment of the EDC‐catalyzed peptide formation also provided a good solubility environment for chitosan. This study demonstrated that the disulfide linkage could be designed as a cleavable linker to trigger the release of a conjugate payload under a certain level of GSH concentration, making the cargo drugs accumulated in tumor cells. Xu et al. reported an oxidation and pH‐responsive chitosan‐based nanoparticle that branched with ferrocene moieties for 5‐fluorouracil delivery.^55^ The DDS was prepared by reductive alkylation of chitosan with ferrocenecarboxaldehyde in the presence of NaBH_4_,^56^ and spherical nanoparticle or vesicles were formed via self‐assembly of ferrocene–chitosan at different concentrations in an acid solution. The 5‐fluorouracil was encapsulated through ultrasonication, and this drug payload was control released in the presence of an oxidative agent at low pH conditions due to the layer‐by‐layer electrostatic repulsion and loss of ferrocene moieties (π–π stacking) of the nanoparticles. Of the available oral DDSs for treating digestive disorders, for example, Jing et al. prepared amoxicillin‐loaded chitosan nanoparticles to target the urea transport protein of *Helicobacter pylori* based on mild ionic gelation of ureido‐conjugated chitosan with sodium tripolyphosphate (TPP).^57^ The ureido group was conjugated to the amino and hydroxyl position of chitosan through amidation and esterification with 12‐ureidododecanoic acid, respectively. The in vitro simulation experiment demonstrated that both of the DDSs were inactivated at pH 1.2 but effectively released amoxicillin at pH 6.0 and pH 7.0, which resulted from the destabilization and weakened electrostatic interaction between chitosan derivatives and TPP at higher pH conditions.

Moreover, the multiple amino groups on the backbone of chitosan would be able to perform any amine related conjugations with other molecules, including methacylation.^58^ Methacylation reaction could enhance the durability of adhesive interfaces. Diolosà et al. reported of using methacrylic acid mixed with chitosan to enhance the adhesion durability with the restorative resin (hydrophobic layer) and the dentine (hydrophilic counterpart) for clinical dental restorations. Methacrylated glycol chitosan can also be cross‐linked using UV light with Irgacure 2959 photoinitiator. Carbodiimide chemistry could also be performed with the amine group on chitosan. Rafat et al. reported the use of EDC/NHS‐mediated cross‐linking reaction or hybrid polyethylene glycol dibutyraldehyde/EDC/NHS to combine collagen and chitosan molecules. These collagen–chitosan composite network scaffolds were verified to enhance the mechanical strength and elasticity for corneal tissue regeneration.^59^ As is well established, unique properties of chitosan make it capable of therapeutic delivery for various application sites including oral, ocular, nasal, vaginal, buccal, parenteral, and intravesical drug delivery.^48^

### 2.2 Alginate‐Based CRS

**Figure 3:**
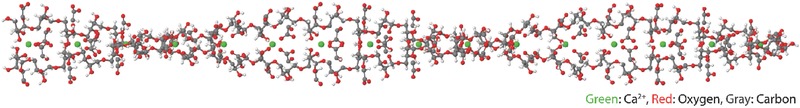
Molecular structure of calcium–alginate junction zone through ionic cross‐linking.^65^

Alginate refers to linear anionic polysaccharides derived from brown algae and bacteria, consisting of repeating units of β‐1,4‐linked d‐mannuronic acid (M) and l‐guluronic acid (G) in varying ratios. Their physicochemical properties (e.g., mechanical flexibility, cross‐linking reactivity, and ionic binding types) have been found to be largely dependent on M/G proportion, the length of block segments, and molecular weight.^40^, ^60^ High contents of G blocks of the alginate are able to form rigid hydrogels with divalent cations such as Ca^2+^, each of which orderly binds to two opposing G blocks, resulting in so‐called egg‐box conformational arrangement (**Figure** **3**).^61^ This is because calcium ions induce chain–chain association. A junction zone (the confirmation structure turn between two adjacent monosaccharide repeat units) was proposed by Grant et al. as confirmed by circular dichroism.^62^ Such facile synthesis approach of these alginates makes them suitable for cell transplantation and tissue regeneration. The semipermeable membrane that alginate and calcium form allows the diffusion of nutrients and therapeutics, maintaining the transplanted cells to grow within the hydrogel, whereas transplanted cell population are protected from the immune systems, which are less immunogenic compared to free cell injection.^63^ Oppositely, using high M block content of the alginates has been found to be less adhesive and exhibits immunostimulatory activity.^64^ This was because M–alginate contained higher level of polyphenol, endotoxins, and proteins compared to G–alginates without additional purification steps before applying in vitro and in vivo. Orive et al. also suggested that purification of alginate by free‐flow electrophoresis would reduce the total impurity content, without provoking foreign body reactions.^38^ This requires high processing and purification standard from alginate industry. The ionically cross‐linking reaction usually occurs by exchanging sodium ions from G blocks with multivalent cations (i.e., Ca^2+^). Therefore, a higher percentage of guluronic acid of the alginate type corresponds to a tighter ionically cross‐linking network, which results in a prolonged release profile.^65^ The mild gelation method has enabled wide application of this type of particles in delivery drugs, plasmid DNAs (pDNAs), growth factors, or even live cells.^37^ The formation of ionic cross‐linking is reversible—that is, adding chelating agent (i.e., ethylenediaminetetraacetic acid) to the already formed nanoparticles will destabilize the cross‐link network, causing particle degradation.

**Figure 4:**
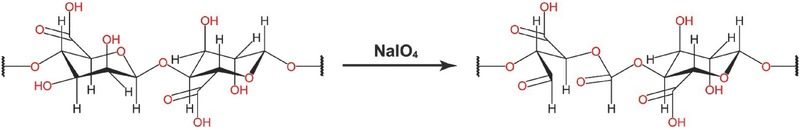
Schematic representation of the chemical synthesis of oxidized alginate.

Alginates have been increasingly employed as a favorable delivery nanoplatform for biomacromolecules and a wide variety of other substances (e.g., growth factors,^40^ cytokines,^37^ doxorubicin,^65^ paclitaxel (PTX),^66^ DNA,^67^ RNA)^68^ to achieve controlled release by varying pore size of alginate hydrogels^69^ as well as by cleavable chemical conjugation using active hydroxyl and carboxyl groups in the polymer backbone.^40^ This is also one of the most mucoadhesive polymers applied in tissue engineering applications.^70^ The most popular routes of conjugation are either to form an amide bond by an EDC/sulfo‐NHS or DCC/DMAP reaction in either aqueous or organic solvent.^71^, ^72^ The water‐based reaction is capable of directing bioconjugation without prior organic solvent dissolution, and the excess of reagent and byproducts can be easily removed by dialysis or gel‐filtration. In order to make alginate soluble in organic solvent, Pawar and Edgar reported a strategy to dissolve tetrabutylammonium salts of alginic acid in polar aprotic solvents containing tetrabutylammonium fluoride,^73^ which is able to react with alginates homogeneously in organic solvents, such as dimethyl sulfoxide and dimethylformamide (DMF). In addition, it has also been reported that hydroxyl groups can be oxidized to form alginate aldehyde with more reactive groups and a faster degradation profile.^74^, ^75^ Since the periodate oxidation of alginate cleaves the carbon–carbon bond of the cis‐diol group in the urinate residue (**Figure** **4**), which changes the chain conformation, it promotes the hydrolysis of alginate in aqueous solutions.^74^ Numerous alginate conjugates have been reported; we now present some recent examples of alginate‐based CRSs.

Feng et al. described redox‐sensitive alginate nanogels for intracellular delivery of doxorubicin (DOX). The nanogels were prepared via in situ cross‐linking of the alginate and the coupling agent cystamine through carbodiimide chemistry and a miniemulsion method.^76^ DOX was encapsulated into nanogels by exploiting the electrostatic interactions between the cationic DOX and the anionic alginate. In one study, vascular endothelial growth factor A (VEFGA)‐encapsulated alginate microsphere with the incorporation of a cyclic arginylglycylaspartic acid (cRGD) peptide and PEG moieties was developed.^72^ This alginate‐based microsphere was designed for receptor‐mediated intracellular delivery and release of the vascular endothelial growth factor A (VEGFA) in primary human mesenchymal stem cells (MSCs) to regulate osteogenic differentiation as a potential therapeutic application. In this study, amine‐terminated PEG oligomers were grafted on alginate through EDC/NHS activation, and cRGD–alginate conjugation included two steps. First, NH_2_–PEG–SH was reacted with 2,2′‐dithiodipyridine to generate pyridine modified NH_2_–PEG. Then, the modified NH_2_–PEG–pyridine was coupled to alginate through carbodiimide chemistry. Another interesting example of alginate‐based oxidation‐responsive delivery system was formed by the conjugation of deferoxamine onto alginate aldehyde via a Schiff‐base reaction and followed by reduction.^77^ These conjugates were explored for their removal of excess iron from the body. It was expected that the alginate–deferoxamine conjugates could protect deferoxamine from metabolism by globulin during circulation and release the active deferoxamine at target sites by local oxidative stress status.

**Figure 5:**
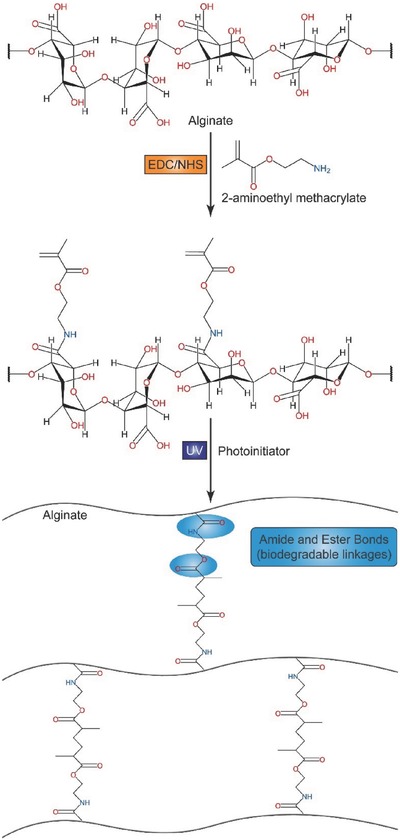
Schematic representation of the chemical synthesis of methacrylated alginate and photo‐cross‐linking of methacrylated alginate. Reproduced with permission.^79^ Copyright 2009, RSC.

In addition, the hydroxyl group of alginate can be reacted with methacrylic anhydride via esterification, which can then be cross‐linked upon exposure to long‐wavelength UV light in the presence of a photoinitiator.^78^ Jeon et al. developed a protocol using 2‐aminoethyl methacrylate to react with the carboxyl group on alginates (**Figure** **5**), providing alternatives to design alginate‐based photosensitive materials.^79^ Jeon et al. also suggested that photo‐cross‐linked oxidized methacrylated‐alginate hydrogels can enhance cell adhesion and spreading compared to those prepared with nonoxidized alginate,^80^ since the free aldehyde group can bind to amines present on cell surface proteins or extracellular matrices.^80^, ^81^ These above strategies for alginate modification provide versatile functionalities for the delivery of therapeutics in a controllable manner and show potential with extensive implementation in the development of innovative DDSs.

### 2.3 Hyaluronic Acid (HA)‐Based CRS

Hyaluronic acid, also known as hyaluronan, is a naturally occurring linear polysaccharide that consists of repeating disaccharide blocks of d‐glucuronic acid and *N*‐acetyl‐d‐glucosamine joined by a glycosidic linkage. It was first isolated from the vitreous humor of bovine eyes by Meyer and Palmer.^82^ Later, it was found to ubiquitously exist in the extracellular matrix of most neural and connective tissues.^83^ Due to the presence of the carboxyl group on each of the glucuronic acid units, HA is naturally negatively charged, which allows it to absorb a large amount of water and swell up to 1000 times its solid dimensions.^38^ The swelling behavior allows the release of drug molecules in a controlled manner.^84^ In the field of tissue engineering, the hydrophilic and viscoelastic properties of HA can not only reduce the friction of the joints but also provide a cushion effect for surrounding tissues.^83^ In addition to its biodegradable and noncytotoxic features, HA has also been considered to be nonimmunogenic and have anti‐inflammatory properties that depend on its molecular weight.^85^ HA is regularly involved in the regulation of angiogenesis, inflammatory, fibrosis, and cancer‐promoting processes.^86^ Moreover, HA can also serve as a targeting molecule that specifically binds to some cell surface receptors including CD44 and receptor for HA‐mediated motility.^12^, ^87^ Owing to its inherent bioactive nature, HA is widely applied as a targeting carrier for delivering therapeutics to tumor tissues,^88^ and as important building blocks for tissue engineering and regenerative medicine,^89^ as well as a common ingredient in cosmetic dermatology.^90^

Similar to alginate, the available hydroxyl and carboxyl groups on HA are commonly used for conjugation via methacrylation^91^ and carbodiimide reaction,^91^ respectively. Methacrylated HA can be photo‐cross‐linked using either ultraviolet (UV) radiation^91^ or visible light.^91^ The distinctive acetamide group (—NHCOCH_3_) of HA is available for deacetylation with the presence of hydrazine sulfate to restore the amine group, which can then undergo amidation reactions for further modification.^92^ Due to the unique and valuable physicochemical property of HA, researchers have been able to design and modify the HA to obtain new specific features for therapeutic delivery in controllable manner. Some recent examples are now given.

Hulsart‐Billström et al. demonstrated a two‐component hydrazide‐modified HA hydrogel‐based adhesive scaffold for bone regeneration through the enzymatic release of active bone morphogenetic protein (BMPs).^93^ In this report, HA–aldehyde (HA–al), HA–hydrazide (HA–hy), and HA–bisphosphonate (BP)–hydrazide (HA–BP–hy) derivatives were used as starting materials, which were obtained from the carbodiimide‐mediated amide coupling of HA carboxyl groups at the carbazate terminal of the reagent. Subsequently, the HA–al solution was mixed with BMP‐2 containing solution of HA–BP–hy or HA–hy to formulate hydrogels encapsulating BMP‐2. The positively charged BMP‐2 can be electrostatically trapped in a negatively charged HA–BP hydrogel, and sustainably released through enzymatic digestion. Moreover, the BP functional group promoted the attachment of the cell to the surface of the HA hydrogel due to the additional Ca^2+^‐mediated linkages. In another example, Baier et al. developed GSH responsive HA‐based nanocapsules by using Cu(I)‐catalyzed “click” reaction polymerization of azide‐functionalized HA and disulfide functionalized dialkyne at the oil‐in‐water miniemulsion droplet interface.^94^ The encapsulated sample dye was released after cellular uptake through the cleavage of disulfide bridges with the presence of GSH in the polytriazole shell of HA nanocapsules.

Fan et al. described a cationic liposome–HA–PEG hybrid nanopolyplexes (NPs) for intranasal vaccination with subunit antigens.^95^ It was composed of a positively charged 1,2‐dioleoyl‐3‐trimethylammonium propane liposomes with incorporation of negatively charged l‐cysteine modified HA (HA–SH) and was further decorated with thiolated PEG via the thiolation of the HA–SH layer on the outer shell of the NPs. This study demonstrated that the F1‐V antigen and monophosphoryl lipid A (MPLA) encapsulated liposome–HA–PEG hybrid NPs could serve as a potential vaccine delivery platform with enhanced biocompatibility, stability, and controlled release for intranasal vaccination against infectious pathogens. Another example of liposome–HA hybrid NPs was reported by Li et al.^96^ The HA–DOX encapsulated liposome was fabricated via two step electrostatic interactions. First, the hydrophobic core of DOX was formed with the presence of soybean oil, and then the HA‐based nanopolyplexes (HA‐NPs) were prepared by ion‐pairing between HA (negative charge) and DOX cores (positive charge). Second, the as‐synthesized HA‐NPs were further encapsulated in liposomal carriers to afford the sustained‐release of DOX by selectively targeting CD44‐positive tumor cells in vivo. Moreover, electrostatic interactions were also employed for targeted gene delivery. Liang et al. prepared a self‐assembled ternary complex consisting of pDNA, branched polyethylenimine (B‐PEI), and HA–epigallocatechin gallate (EGCG) for CD44‐targeted delivery of nucleotides.^97^ This DDS was first stabilized by self‐assembly of pDNA and B‐PEI via electrostatic interactions, and the resulting positively charged pDNA/B‐PEI complexes were subsequently coated by HA–EGCG conjugates. These ternary complexes processed an efficient targeting cancer cell transfection due to the CD44‐targeting ability, and B‐PEI induced endosome escape and the strong nucleotide‐binding affinity of catechin.

Recently, Zhong et al. formed endosomal pH‐activatable micelles via the self‐assembly of HA‐*b*‐dendritic oligoglycerol block copolymer (HA–dOG–PTX).^98^ The azide‐terminated [G1.0] dendritic oligoglycerol conjugate (N_3_–dOG) was first reacted with succinic anhydride to form N_3_–dOG–COOH, which was then converted to N_3_–dOG–vinyl ether by adding 2‐chloroethyl vinyl ether under a nitrogen atmosphere. PTX was grafted to N_3_–dOG–vinyl ether via an acetal linkage through the acid‐catalyzed reaction of the 2′‐hydroxyl group in PTX with vinyl ether terminates. HA–alkynyl was synthesized by the reductive amination of HA terminal aldehyde with propargylamine, and conjugated to N_3_–dOG–PTX through a click reaction. The resulting conjugate (HA–dOG–PTX) was then self‐assembled into prodrug micelles, which demonstrated higher payload, CD44 targetability and pH‐response capabilities than the free drug.

The formation of bioreducible HA composites has also been reported by disulfide cross‐linking HA to other molecules.^99^, ^100^ Han et al. described the fabrication of DOX‐loaded bioreducible HA nanoparticles (DOX–HA–ss‐NPs) with a redox‐responsive drug release profile and improved antitumor efficacy in the treatment of SCC7 tumor in a xenograft model.^100^ First, alkyl‐terminated HA was prepared by reductive amination, as aforementioned, and 2‐(pyridyldithio)‐ethylamine (PDA) was conjugated with alkyne–HA through EDC‐mediated amidation. Second, another building block azide‐functionalized polycaprolactone (PCL–N_3_) was synthesized via the ring‐opening polymerization of caprolactone, followed by tosylation of PCL–OH, and conversion into the azide group by a nucleophilic displacement reaction.^101^ Finally, the PDA‐conjugated HA–*b*–PCL copolymer was obtained via Huisgen cycloaddition between PCL–N_3_ and alkyne–HA–PDA. The resulting shell cross‐linked HA–ss‐NP was formed by the dithiothreitol (DTT) catalyzed cross‐linking of the PDA‐conjugated HA–*b*–PCL, and loaded with DOX through an emulsion method. Similarly, a bioreducible core‐cross‐linked HA micelle (CC‐HAM) for anticancer therapy was reported in another study of Han et al.^99^ In this case, the building block of HA‐based core‐cross‐linked polymeric micelle was also prepared by Huisgen cycloaddition between alkyne–HA and azide terminated poly(pyridyl disulfide methacrylate) [P(PDSMA)]. The P(PDSMA) was synthesized by polymerization of the monomer 2‐(pyridine‐2‐yldisulfanyl) ethyl methacrylate with 2‐azidoethyl‐2‐bromo‐2‐methylpropanoate. The DOX‐loaded CC‐HAM was formed via the self‐assembly of amphiphilic HA–*b*–P(PDSMA) with the presence of DTT. In a related work, Zhong et al. described the redox‐sensitive HA–l‐lysine methyl ester‐lipoic acid (HA–Lys–LA) conjugates for active targeting delivery of DOX to the drug resistant CD44 positive human breast tumor in vitro and in vivo.^102^ The l‐lysine methyl ester was first grafted to HA via EDC/NHS activation. The amino group of intermediate (HA–Lys) was then conjugated with the carboxylic group of lipoic acid via DCC/DMAP‐mediated amidation. The resulting cross‐linked NPs were obtained by self‐assembly of HA–Lys–LA conjugates with the presence of DOX and a catalytic amount of DTT. Among its applications, the broad spectrum of options for HA chemical modifications can be applied to achieve a specific targeting and long‐lasting delivery of various therapeutics, including protein, peptides, and small molecule drugs.^103^

### 2.4 Dextran‐Based CRS

Dextran is a group of branched anhydroglucose polymer composed of alpha‐1‐6 glucose‐linked glucan with side chains alpha‐1‐3 linkages attached to the backbone units of dextran. They are produced from the fermentation of sucrose by certain lactic acid bacteria. Dextran holds several physicochemical advantages regarding its superb water solubility, surface resistance to nonspecific protein adsorption,^104^ and ease of chemical derivatization, which make it suitable to be modified for therapeutic delivery. The abundant microbial enzyme dextranases, which could enzymatically digest dextran in colon tissue, making dextran suitable as potential nanovehicles for colon specific drug delivery.^105^ Similar to other types of polysaccharides, the properties of dextrans are strongly dependent on their structure including molecular weight and degree of branching. According to the backbone structure of dextran, the hydroxyl groups of dextran units and the terminal aldehyde groups of dextran are two common reaction sites for chemical conjugation. Alternatively, two additional aldehyde groups can also be established from the periodate oxidation of dextran.^106^

In one example, intracellular acidity‐sensitive dextran–DOX conjugates (Dex–O–DOX) were prepared by employing an oxime click reaction between the amino group in DOX and the terminal aldehyde group of dextran,^107^ which afforded the pH‐triggered intercellular release of DOX via the breakdown of the Schiff‐base linkage. The in vitro and in vivo evaluation revealed that Dex–O–DOX increased antitumor activity and reduced toxicity compared with the reduction type Dex–*b*–DOX. Zhu et al. developed a lysosome‐targeted acidity‐responsive nanomicelles (Dex/Chol–PBA) through self‐assembling dextran and phenylboronic acid modified cholesterol.^108^ First, phenylboronic acid was coupled with cholesterol by adding *N*‐methylimidazole.^109^ Then, Dex/Chol–PBA nanomicelles were prepared by dynamic self‐assembly between Dex and Chol–PBA via pH‐dependent phenylboronate linking. Through the in vivo/in vitro evaluation work, it was clearly confirmed that DOX‐loaded Dex/Chol–PBA nanomicelles exhibited an efficient cholesterol‐assisted cellular uptake, lysosome‐acidity induced drug liberation, and excellent safety profile.

Recently, Cao et al. combined two types of stimuli‐sensitive dextran conjugated prodrugs for combinatory cancer therapy.^110^ They first prepared a dextran propargyl carbonate (dex—C≡C) by activating propargyl alcohol with the presence of carbonyl diimidazole.^111^ Later, the activated compounds were coupled with a dextran backbone through the formation of hydrolyzable carbonate esters. Subsequently, the dex—C≡C was conjugated with azide‐functionalized redox‐sensitive (disulfide bond) camptothecin derivative (CPT—ss—N_3_) via Huisgen cycloaddition, which resulted in the formation of the prodrug Dex—ss—CPT. For another pH‐sensitive dextran–hydrazone–doxorubicin (Dex—hyd—DOX) prodrug, a similar synthetic approach was applied to couple the azide‐functionalized pH‐responsive (hydrazone bond) DOX derivative (DOX—hyd—N_3_) with dex—C≡C. The preclinical evaluation of the combinatory therapy using Dex—ss—CPT and Dex—hyd—DOX micelles demonstrated significant anticancer activity by passively targeting tumor microenvironment and optimizing the synergistic effect molar ratio of DOX and CPT. Another interesting case for the redox‐responsive DOX carrier for triggered drug release using the GSH reducible dextran–Pt(IV) conjugate was reported by He et al.^112^ They first prepared a carboxyl group functionalized platinum(IV) complex,^113^ and then they synthesized the amphiphilic dextran–Pt(IV) conjugate based on the esterification between the carboxyl group of the Pt(IV) complex and the hydroxyl group of dextran. The DOX was further encapsulated into the hydrophobic center of dextran–Pt(IV) conjugate through self‐assembly. In cancer cells with presence of reductants including GSH and ascorbate, Pt(IV) moieties were reduced to the active Pt(II) form and cleaved from dextran side chains to induce the disruption of the conjugate structure, leading to the rapid liberation of dual drugs.^112^

### 2.5 Cyclodextrin (CD)‐Based CRS

**Figure 6:**
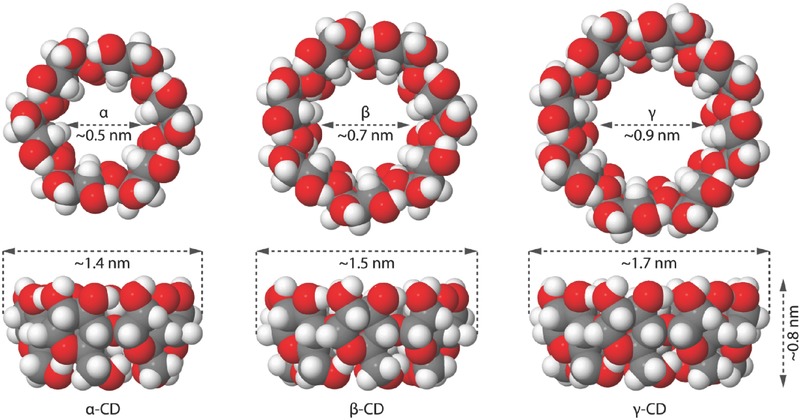
Schematic representations of molecular structure and geometric dimensions of α, β, and γ‐cyclodextrin.

CDs are cyclic oligosaccharides constructed by 6 (α) or 7 (β) or 8 (γ) glucopyranose units through α‐1,4‐glycosidic linkages and possess a cage‐like structure with a hydrophobic interior cavity and a hydrophilic exterior surface (**Figure** **6**). They are synthetic compounds obtained from the enzymatic hydrolysis of starch by *Bacillus macerans*.^114^ The extraordinary trapping ability leads to a host–guest interaction between hydrophobic guest species and the interior cavity of CDs, given the modified physicochemical properties of the guest molecules in biological milieu. Since they are generally safe, inexpensive, water soluble, and easily functionalized, CDs have been intensively explored for therapeutic delivery. However, due to the relatively small cavity size of CDs, only limited number of molecules can be encapsulated for drug delivery; thus, the CD‐conjugated amphiphilic nanoformulation in the form of host–guest complexes has been increasingly developed in recent years.^115^, ^116^ In addition, supramolecular hydrogels that utilize the interactions between a CD host and guest polymers to form inclusion complexes have attracted considerable attention to the tissue engineering field.^117^

**Figure 7:**
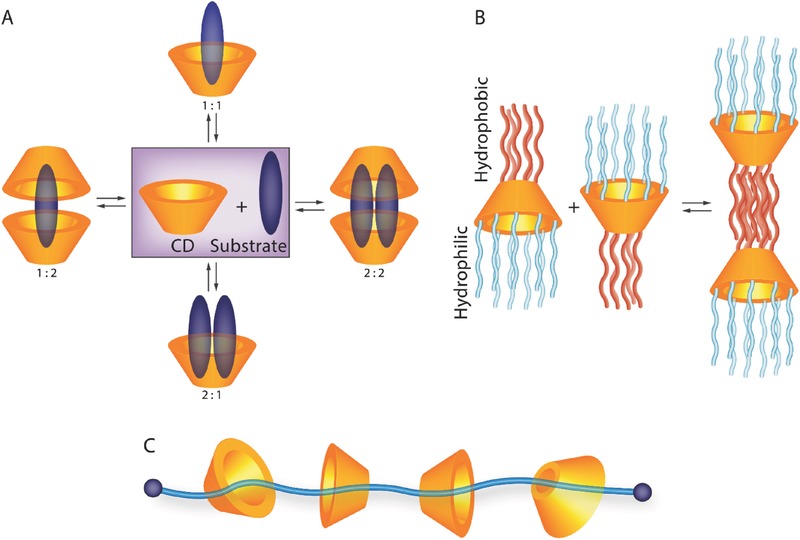
Schematic illustration of three types of CD‐based assemblies. A) Substrate–CD inclusion complexes. B) Amphiphilic CD conjugates. C) CD‐based pseudo‐polyrotaxane (PPR).

On the basis of construction for CD‐based assemblies, there are several exciting possibilities to design host/drug complex systems for therapeutic delivery (**Figure** **7**), including substrate–CD inclusion complexes (substrate/CD ratio: 1:1, 1:2, 2:1, and 2:2), amphiphilic CD conjugates, and CD‐based pseudo‐polyrotaxane (PPR). Recently, there is a great trend of employing synergistic interactions to constitute CD‐based self‐assemblies that result from the combination of various intermolecular forces, such as hydrophobic, electrostatic, covalent, and hydrogen binding.^118^, ^119^ Besides, their formation and dissociation of CD‐based self‐assemblies are designed to be sensitive to biological milieu variations, for instance, pH value, temperature, redox, and enzyme.^116^, ^120^ CDs are Food and Drug Administration approved cyclic macromolecules for application in food, cosmetics, and pharmaceuticals. By taking full advantage of these features, CDs can be utilized as molecular valves to control the conformational change of the supramolecular system for the release of therapeutic payloads. In this regard, we will highlight recent advances in the chemical modification and bioapplication of CD‐based CRS.

#### 2.5.1 Substrate/CD Inclusion Complexes

Nobusawa et al. described a pH‐sensitive fullerene (C_60_)/6‐amino‐γ‐CD (ACD) inclusion complex for photodynamic therapy.^121^ First, the protonatable primary amino moieties were grafted on the primary face of γ‐CDs through the reduction of intermediate azide modified γ‐CDs by employing triphenylphosphine (PPh_3_) in DMF.^122^ Subsequently, each C_60_ was hydrophobically encapsulated in two γ‐CDs under neutral pH conditions. The in vitro evaluation of photodynamic therapy for HeLa cells demonstrated that the protonation of the amine groups of C_60_/ACDs at slightly acidic conditions led to the electrostatic repulsion of the wide rim, followed by the shrinkage of narrow rim, thus triggering the release and aggregation of C_60_ surrounded by protonated ACDs.^121^

Besides drug inclusion, CDs were also utilized as a key component for preparing stimuli‐induced supramolecular vesicles. Recently, Nayak and Gopidas designed and synthesized unusual supramolecular vesicles through the spontaneous self‐assembly of β‐CD/adamantane (AD)‐based bis‐inclusion complex (β‐CD∈AD–AD∋β‐CD).^123^ Regarding the β‐CD∈AD–AD∋β‐CD system, the AD–AD molecule behaved as the amphiphilic bridge, which consisted of an ethylenedipyridine core and two adamantane moieties on both ends. The hydrophilicity is attributed to positively charged pyridinium residue, and adamantane moieties acted as hydrophobic head. In contrast, two β‐CDs served as the hydrophilic cap that accommodates the adamantane ends to form the bis‐inclusion complex. The AD–AD molecule was synthesized by the alkylation of amine at ethylenedipyridine with adamantyloxy ethyl bromide.^123^ DOX loaded β‐CD/AD vesicles showed potential application for controlled release of drug by addition of a competitive inclusion binder such as adamantane carboxylate that simultaneously disrupted the vesicles. Similarly, Ma et al. developed a simple supramolecular self‐assembled binary vesicle based on tyrosine/β‐CD inclusion complexes.^124^ Since the primary rim of the β‐CD molecule has a stronger hydrophilic properties than the carboxyl group of tyrosine exposed at the exterior part of secondary rim of β‐CD, the tyrosine/β‐CD amphiphiles self‐assembled into binary vesicles that were driven by hydrophobic–hydrophobic interaction.^124^ The vesicles can respond to multiple exogenous stimuli, by disrupting the conformation of the vesicles via competitive guest molecules (1‐hydroxyadmantane) and copper ions. The 1‐hydroxyadmantane entered the cavity of β‐CD to replace tyrosine, or the copper ions coordinated with tyrosine molecules to form stable metal–organic complexes.^124^

#### 2.5.2 Multifunctional Amphiphilic CD Conjugates

One of the prominent strategies in designing CD‐based therapeutic delivery systems is multifunctional conjugates that integrate stealth effects, active targeting, stimuli‐response, and imaging monitoring to provide greater therapeutic improvement. For example, the most remarkable CD‐based conjugate formulations for gene delivery is β‐cyclodextrin–polyethylene glycol copolymer (β‐CDP) polyplexes and its derivatives, which were developed by Davis and co‐workers. In their early study, pDNA encapsulated PEGylated β‐CDP polyplexes were designed and prepared through electrostatic interaction between positively charged β‐CDP polyplexes and negatively charged pDNA under physiological conditions^125^ The β‐CDP was synthesized via the cross‐linking reaction of dimethyl suberimidate and amine‐functionalized β‐CD. Adamantane–PEG (AD–PEG) conjugates were self‐assembled with polyplexes to form Ada–PEG/β‐CDP inclusion complexes for prolonged circulation. In another study, they modified the AD–pep–PEG with galactose to obtain the active targeting ligand AD–pep–PEG–gal, which was decorated on pDNA loaded PEGylated β‐CDP polyplexes for selective binding to hepatocytes through overexpressed asialoglycoprotein receptors.^126^ In 2007, they reported similar CDP polyplexes by replacing galactose with transferrin (AD–PEG–transferrin) for targeted delivery of siRNA to transferrin‐receptor‐upregulated HeLa cells^127^ as well as to metastatic Ewing's sarcoma in a mouse model.^128^ They further evaluated the safety profile with escalating intravenous doses of siRNA containing AD–PEG–transferrin polyplexes in nonhuman primates.^129^ Ultimately, it entered a clinical trial (under the name CALAA‐01) for RNA interference (RNAi) in human tumors.^130^, ^131^

Another prominent example of CDP nanoparticles is CRLX101, which was designed to address the poor drug solubility, insufficient chemical stability in physiological environments, and off‐target toxicity of CPT.^132^ CPT is a topoisomerase I inhibitor with remarkable anticancer activity, which was interventionally linked to the repeating units of CD and PEG blocks via a glycine linker. Such arrangement could lead to the self‐assembly of the copolymer into 20–60 nm sized particles due to the host/drug interactions between adjacent CDP strands.^133^ The resulting CRLX101 possessed neutral surface charge and with PEG blocks exposed to the outer layer. In addition, the CPT can be activated at target sites through the cleavage of the glycine linker that was medicated by both the base‐catalyzed and enzymatic hydrolysis of the ester group.^132^ Both preclinical and clinical studies demonstrated improved solubility and an extended circulation time as well as reduced toxicity of CPT; these studies also exhibited enhanced therapeutic efficacy of CPT.^132^, ^133^, ^134^, ^135^

Namgung et al. described self‐assembled polyplexes that were prepared by a multivalent inclusion complexation between a polymer–β‐CD conjugate (pCD) and a polymer–paclitaxel (pPTX) with active targeting and controlled release of PTX via in vivo enzyme‐degradation and the hydrolysis of ester linkages.^136^ First, the β‐CD was grafted on poly(isobutylenealt‐MAnh) through esterification of maleic anhydride units with a single‐selectively deprotected hydroxyl group of β‐CDs. Second, the 2′‐hydroxyl group of PTX was preferentially reacted with anhydride groups of the poly(methyl vinyl ether‐alt‐MAnh) to form pPTX.^136^ Next, the FCR‐675 fluorescent dye and targeting ligand, AP‐1 peptide were conjugated to pPTX by an amine–anhydride reaction and a PDA linkage, respectively.^136^ The FCR‐675/AP‐1 grafted pPTX was then self‐assembled with pCD through a multivalent inclusion complexation, and the resulting polyplexes were found to have higher stability and solubility than that of the monovalent PTX‐β‐CDs. They also exhibited the stimuli‐responsive PTX release and potential tumor targeting through passive and active targeting mechanisms.

Wajs et al. have recently reported stable redox or light responsive hollow nanocapsules based on ferrocene/β‐CD or azobenzene/α‐CD‐decorated dextran polymers.^137^ Both kinds of nanocapsules were prepared through layer‐by‐layer self‐assembly of host/guest polymers that deposited on the surface of Au colloid templates. The Au nanoparticles were initially coated by thiolated β‐CD or α‐CD dextran polymers (host), followed by the deposition of ferrocene or azobenzene functionalized dextran polymers (guest) on the outer layer via host/guest interaction. Finally, they removed the oxidative core to obtain the hollow nanocapsules.^137^ In this report, the authors demonstrated that Rhodamine B can be encapsulated and released via a reversible one‐electron redox process (ferrocene‐based nanocapsules) and UV‐light irradiation (azobenzene‐based nanocapsules) by the altering wall permeability of the inclusion complex.^137^

#### 2.5.3 CD‐Based PPR

CD‐based PPRs are noncovalently interlocked supermolecular architectures that are comprised of linear polymer components (guests) and encircled by CD components (hosts), and they are advancing rapidly in the area of stimuli‐responsive materials due to their unique features.^138^ Using similar mechanisms, Dandekar et al. developed a cationic α‐/β‐CD‐based polyrotaxane, which can condense nucleic acids into nanoplexes for in vitro gene delivery.^139^ The CD polyrotaxane was obtained by subsequent incubation of amine functionalized β‐CD and α‐CD with ionene‐6,10 polymer, as the CD rings were threaded over the polymer chain with temperature activated noncovalent interactions.^139^, ^140^ The nanoplexes were then formulated with pDNA and siRNA via electrostatic interaction. The cellular investigations demonstrated that their nanoplexes could successfully overcome the endosome degradation with low cytotoxicity for intracellular gene delivery.

In one study, we developed self‐healing, thermoresponsive host–guest inclusion complexes (i.e., Pluronic F108 incorporated alginate‐*graft*‐β‐cyclodextrin) for cell transplantation and drug delivery.^71^ To synthesize alginate‐*graft*‐β‐cyclodextrin, p‐toluenesulfonyl (TosCl) chloride was first reacted with β‐CD to yield β‐CD–TosCl. Then 1,6‐hexanediamine (HDA) and ethylenediamine (EDA) were reacted with β‐CD–TosCl to obtain β‐CD–HDA and β‐CD–EDA, followed by amide bond formation with alginate via carbodiimide chemistry. Finally, the resulting product alginate‐*graft*‐β‐CD was self‐assembled with the difunctional guest molecule Pluronic F108 through a host/guest interaction. This because the hydrophobic moieties of Pluronic F108 is held within the cavity of β‐CD. Based on these unique intermolecular interactions, such supramolecular inclusion complex exhibits shear‐thinning properties and affords excellent thermal‐responsive behavior to the injectable hydrogel. Such shear‐thinning hydrogel flows similar to low‐viscosity fluids under shear stress during injection. However, as soon as the fluid comes out the needle, hydrogel recovers by itself without additional trigger factors, such as UV light. Shear‐thinning hydrogels have been extensity studied in various disease model and even 3D printing polysaccharides.^26^, ^141^

Recently, Badwaik et al. reported three cholesterol terminated Pluronic (F‐127, L‐35, and L‐81) cationic polyrotaxanes (PR^+^) threaded with *N*,*N*‐dimethylaminoethylamine (DMEDA)‐functionalized 2‐hydroxypropyl (HP)–β‐CD for siRNA delivery.^142^ DMEDA was conjugated to HP–β‐CD via a carbonyldiimidazole‐mediated coupling reaction.^143^, ^144^ The HP–β‐CD units were first threaded onto the Pluronic copolymer backbone, followed by introducing tris(2‐aminoethyl)amine at both ends, and finally end‐capping the branched diamine termini with cholesteryl chloroformate.^142^, ^143^ The resulting PR^+^:siRNA formulation was obtained through electrostatic interactions between the PR^+^ and siRNA payload, which exhibited higher performance than Lipofectamine 2000, while maintaining low cytotoxicity and high in vitro stability.^143^

Tamura et al. developed a novel acid‐responsive β‐CD‐based polyrotaxanes for the treatment of Niemann–Pick type C (NPC) disease.^145^ NPC disease is a rare inherited lysosomal storage disorder with mutations in NPC1 and NPC2 genes.^146^ The key feature of the disease mechanism is the accumulation of cholesterol within lysosomes, and it has been found that intracellular cholesterol can be effectively dissolved away through inclusion complexation with HP–β‐CD. However, excessive HP–β‐CD can induce various acute toxicities in animal models;^147^ thus, to overcome the toxic issue, they designed and synthesized a pH‐sensitive polyrotaxanes system comprised of three different components: Pluronic P123 polymer, threading 2‐(2‐hydroxyethoxy)ethyl (HEE)‐functionalized β‐CDs and terminal *N*‐triphenylmethyl (N‐Trt) blocks.^145^ The in vitro evaluations demonstrated that the acid‐responsive β‐CD‐based polyrotaxanes can be internalized into cells through endocytosis and spontaneously dissociated the HEE–β‐CDs under acid environments.^145^, ^148^ When polyrotaxane occupies the β‐CD cavity by the polyrotaxane structure, it will not only mask the cytotoxicity by preventing the extraction of cholesterol in membranes, but also provide improved therapeutic efficacy by three orders of magnitude over HP–β‐CD.^145^

## 3 Preparation Approaches

The advanced understanding of material chemistry and engineering techniques facilitates multiple strategies to fabricate polysaccharide‐based DDSs. In this section, we discuss the chemistry basics associated with different cross‐linking forces within polysaccharide systems and the engineering techniques used to fabricate polysaccharide‐based DDSs.

### 3.1 Intra‐ and Intermolecular Forces in Polysaccharide Systems

#### 3.1.1 Covalent Cross‐Linking

**Figure 8:**
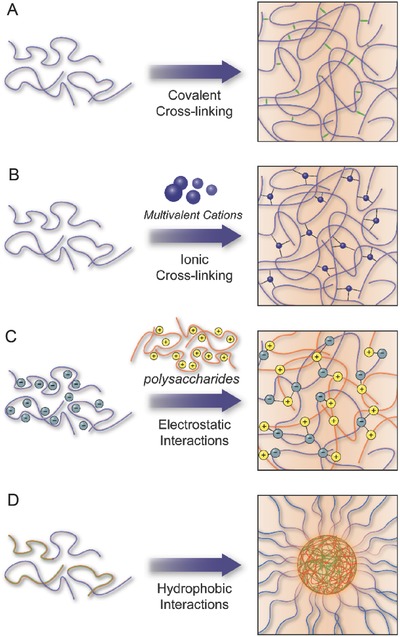
Schematic illustration of different intra‐ and intermolecular forces in polysaccharide systems. A) Covalent cross‐linking. B) Metal–polymer coordination. C) Electrostatic interactions. D) Hydrophobic interactions.

To maintain the network of polysaccharide NPs that avoid dissolution of the hydrophilic polymer chains/segments into the aqueous phase, chemical cross‐linking is usually performed while maintaining the biodegradability of the materials (**Figure** **8**A). In chemically cross‐linked NPs and gels, covalent bonds are established between functional groups of polymeric chains or are mediated by covalent cross‐linking molecules with at least two active moieties.^9^ The chemical linkages in the matrix structure are usually designed either to be biodegradable or stimuli‐responsive under specific endogenous and exogenous conditions.^20^, ^21^, ^149^ Although the covalent cross‐linkages are the major driving force, other noncovalent forces (e.g., hydrogen bonding and hydrophobic interactions) could also be involved, depending on the types of polysaccharides and chemical modifications employed. In general, labile bonds including peptide bonds (carbodiimide‐mediated reactions), ester bonds (anhydride‐mediated esterification), and disulfide bonds (oxidation of the thiol groups) commonly facilitate the intramolecular cross‐linking of the polysaccharide network.^21^, ^150^ In the previous paragraph, we discussed the methacylation reaction and its function in photocross‐linking reactions, which is an interesting example of covalent bonds being applied to design polysaccharide NPs.

#### 3.1.2 Metal–Polymer Coordination

In contrast to covalent cross‐linking, metal–polymer coordination forms stronger bridges between polysaccharide chains through coordinate–covalent bonds (chelation) between metal cations (e.g., calcium, copper, iron, zinc) and negatively charged ligand moieties of polysaccharides (Figure 8B).^9^, ^151^ This intramolecular force enables the reversible and facile formation of metal–polysaccharide nanocomposites,^152^ such as hydrogels with variable physicochemical properties that depend upon the size and the valence of anionic metals, as well as degree of chemical modification and concentration of the polysaccharide.^152^, ^153^, ^154^ In addition, metal–polymer coordinates are generally pH sensitive, which is favorable for controlled drug release, although this may also cause instability of the cross‐linked network.^65^ To date, alginate is a well‐known example of polysaccharide that can be cross‐linked by metal–coordinate interactions by exchanging sodium ions from the guluronic units with divalent cations, mainly the Ca^2+^ ions.^155^ These calcium ions are coordinated to the hydroxyl and carboxyl groups of four α‐l‐guluronic acid units from two adjacent chains of the alginates,^156^ and as a result, the hydrogel network with a so‐called “egg‐box” structure is formed.^157^ The alginate gel beads can be prepared at room temperature and physiological pH; thus, they are widely used for the immobilization of living cells and the controlled release of a variety of proteins.^158^

#### 3.1.3 Electrostatic Interactions

In addition to anionic polysaccharide being coordinately cross‐linked with metallic ions, polyelectrolyte complexes (PECs) can also be obtained by electrostatic interactions between oppositely charged polysaccharide and polyelectrolytes in solution (Figure 8C)^154^, ^159^ PECs provide a reversible and noncovalent physical linkage without using any reactive agents and catalysts for the immobilization of therapeutic payloads. PECs are any positively or negatively charged macromolecules like nucleic acids (e.g., pDNA, siRNA), proteins (e.g., albumin, collagen, gelatin), polysaccharides (e.g., chitosan, hyaluronic acid, alginate), and synthetic polycation and polyanion polymers (e.g., polyethylenimine, polyacrylic acid).^153^, ^160^ The complexation, stability, and physical properties (e.g., permeability, swelling) of PECs are determined by several factors, including the intrinsic properties of PECs (e.g., ionic strength, charge density, molecular weight, flexibility) and physicochemical environment (e.g., temperature and pH of the solution, type of solvent, degree of interaction between PECs and polysaccharides) as well as the order and duration of mixing PECs.^21^, ^149^, ^152^, ^160^, ^161^ Among the existing polysaccharides, chitosan is the most commonly applied cationic polysaccharide to form PECs due to its biocompatible and water‐soluble features,^20^, ^149^ whereas hyaluronic acid,^162^ dextran sulfate,^163^ alginate,^164^ nucleic acids,^165^ and some aspartic acid and glutamic acid‐rich peptides/proteins are used as anionic polyelectrolytes.^166^, ^167^ In addition, anionic polysaccharides can also form PECs with positively charged peptides/proteins, such as polylysine, which is a positively charged peptide that electrostatically combines with alginate to form PEC nanoparticles.^166^

#### 3.1.4 Hydrophobic Interactions

Upon introducing hydrophobic segments onto the hydrophilic polysaccharide chains, amphiphilic copolymers are produced. These copolymers tend to self‐assemble into stable conformations to minimize the free energy by spontaneous formation of hydrogen bonding between the hydrophilic backbone of the polysaccharide and water molecules. Hydrophobic blocks undergo self‐association to form a hydrophobic domain due to the unfavorable interaction with water (Figure 8A).^168^ The amino, carboxyl, and hydroxyl groups present on polysaccharide backbone are the most utilized functional pendant groups to conjugate a wide range of hydrophobic segments, such as cholesterols, fatty acids, bile acids, polyesters, pluronic polymers, poly(alkyl cyanoacrylate), and hydrophobic drugs.^20^, ^21^ Various self‐aggregates that are based on hydrophobized polysaccharides (e.g., hydrogel nanoparticles, micelles, polymersomes, oil in water (O/W) emulsions) can be formed for the controlled delivery of hydrophobic compounds via stimuli responses, such as pH, temperature, and enzyme‐degradation.^168^, ^169^ For clinical translation of controlled drug delivery formulations, a number of parameters including size, solubility, loading capacity, surface charge, physiological stability, and drug release kinetics need to considered, which can be achieved by adjusting the functional group, molecular weight, and concentrations of the hydrophobic block and polysaccharides.^154^, ^170^ Among those amphiphilic copolymers, amphiphilic CDs have gained significance in pharmaceutical formulations to encapsulate hydrophobic drug molecules through their hydrophobic cavity. Recent progress in the development of CD‐based complexation system has inspired the way of supramolecular self‐assembly for drug delivery, and examples have been discussed in the previous section of this review.

### 3.2 Fabrication Methods and Techniques

#### 3.2.1 Emulsification Method

Emulsification is one of the most evolved methods for the preparation of polymeric nanoparticles for research and pharmacotherapy applications. The success of emulsion comes from several attributes, such as optical clarity, ease of preparation, thermodynamic stability, and increased surface area. Phase behavior studies have shown that the size of the droplets is determined by the surfactant phase structure (bicontinuous microemulsion or lamellar) at the inversion point that is induced by either material composition or temperature. The preparation of an emulsified system is generated by mixing two or more immiscible liquids and using mechanical processes, such as stirring or ultrasonication. Generally, depending on the type of liquid that is used for the dispersed and continuous phase, O/W or water in oil (W/O) emulsions can be formed, and multiple emulsions (e.g., W/O/W and O/W/O) can also be achieved to enhance the efficacy of formation of emulsion droplets and to encapsulate drugs with different solubility in different phases. According to the size of the droplets, the emulsions are classified into three main types: a microemulsion is primarily referred to as a thermodynamically stable droplet with size ranging from 10 to 100 nm; a nanoemulsion is characterized by a thermodynamically unstable but kinetically stable feature with droplet sizes mostly between 20 and 500 nm; and a macroemulsion represents a classical emulsion system that often exhibits thermodynamically unstable and weakly kinetically stable behavior with droplet size greater than 1 µm.^171^

Since polysaccharides are usually water soluble, W/O is mostly applicable for the fabrication of polysaccharide‐based nanoparticles. The emulsion‐cross‐linking method was initially applied to the preparation of chitosan nanoparticles for 5‐fluorouracil delivery. In this process, a chitosan aqueous solution was emulsified in toluene, followed by cross‐linking with glutaraldehyde to harden the droplets.^172^ The principle of cross‐linking was based on a Schiff‐base reaction between the aldehydic group of the glutaraldehyde and the primary amines of chitosan,^173^ which formed the inter‐ and intramolecular covalent network to firm up the structure of the chitosan particle. However, there are concerns over the toxicity of the glutaraldehyde used, which compromised the biocompatibility of chitosan‐based emulsions. Therefore, efforts had been made to ameliorate the cross‐linking method. One solution is to replace the glutaraldehyde with biocompatible cross‐linking agents such as glyceraldehyde and genipin.^174^, ^175^ Recently, Song et al. prepared PEG‐modified ultrasmall chitosan nanoparticles as indocyanine green (ICG) carriers with the average size around 5 nm for tumor photothermal therapy in vivo.^175^ An aqueous dispersion of chitosan was added into the microemulsion system consisting of cyclohexane, 1‐octanol, and Triton X‐100, and the mixture was stabilized using ultrasound. The microemulsions were hardened by genipin cross‐linking.^175^ PEG‐modified chitosan–genipin nanoparticles were prepared via the conjugation of succinimidyl carboxymethyl ester (SCM–PEG) on the surface of the nanoparticles, and ICG molecules were subsequently loaded into the nanoparticles using electrostatic interactions.^175^ When irradiated with a NIR laser, cells incubated with CG–PEG–ICG nanoparticles showed cell viability around 15%. The in vivo bioavailability and efficacy of the photothermal therapy effect on the treatment of U87 xenograft tumors by intravenous and intramuscular injection was evaluated, respectively, and the results demonstrated that CG–PEG–ICG nanoparticles exhibited prolonged retention time of ICG in the mice body as well as low toxicity with effective tumor phototherapy (tumor injected with CG–PEG–ICG nanoparticles containing ICG more than 100 µg mL^−1^ (100 µL)).^175^

**Figure 9:**
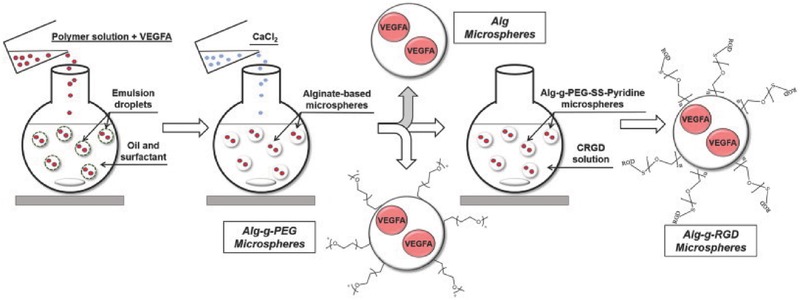
Schematic representation of fabrication of micropsheres via water/oil emulsion. Reproduced with permission.^22^ Copyright 2014, Elsevier.

In addition to covalent approaches, the emulsion‐ionic cross‐linking interaction has also been applied to prepare chitosan microspheres. For instance, Zou et al. reported that sodium TPP, a biocompatible polyanion, was introduced to prepare cross‐linking chitosan microparticles (5–10 µm) for pH‐responsive release of bovine serum albumin (BSA).^176^ The controlled release of BSA was mediated by diffusion via the swelling behavior of chitosan microspheres, which exhibited a higher swelling ratio and was more promising than glutaraldehyde cross‐linked microspheres.^176^ Machado et al. described the preparation of W/O type nanoemulsions of aqueous alginate solutions through the phase inversion temperature emulsification method.^177^ In this experiment, they employed nonionic ethylene oxide oligomers (C_12_E_4_) as a temperature dependent surfactant, which exhibits increased hydrophobicity with rising temperatures. The structure of emulsions could change from O/W to W/O via temperature control. Ionic cross‐linking of the alginate was performed by introducing aqueous CaCl_2_ to the emulsions under stirring, and prepared nanoparticles were collected through addition of excess oil. This method allows the preparation of finely dispersed calcium alginate nanoparticles in the sub‐200 nm range without a large input of mechanical process.^177^ Recently, we have reported utilizing 5% Span 80 in mineral oil as the oil phase with addition of tween 80 as the surfactant and 1% alginate solution to form alginate microparticles cross‐linked by CaCl_2_. The reaction was easily performed on bench top at room temperature, simplifying the previously stated method, but achieving evenly distributed nanoparticles (**Figure** **9**). In addition to the normal alginate solution, we also used a PEGylated alginate for multifunctional microparticles with a similar method. The polymer (alginate or PEGylated alginate)/drug solution was slowly added to biological‐grade mineral oil containing surfactants. CaCl_2_ was added to the system while stirring to cross‐link alginate to from stable microparticles. After the reaction, the particles were washed several times to remove the mineral oil. The obtained particles were spherical in shape with an average diameter of 1–5 µm and can be lyophilized and stored for long‐term application.^22^

#### 3.2.2 Desolvation (Coacervation or Precipitation) Method

The desolvation method is a facile synthetic approach that often involves coacervating or precipitating a polysaccharide matrix in an aqueous solution and forming polymeric micro‐/nanoparticles by addition of desolvating agents, such as salts or alcohols. This process is induced by the competitive binding of desolvating agents to water molecules in a previously formed polysaccharide solution. The surrounding water molecules are consequently dissociated from the polysaccharide micro‐/nanoparticles due to the higher affinity between water and the desolvating agents.^178^, ^179^ A cross‐linking agent is commonly used for further stabilization and adapted for the controlled release of the therapeutic payload.

One significant benefit of the desolvation approach is that usually no heated reaction condition is required, as some encapsulated drug molecules or bioactive agents are thermodynamically unstable.^180^ However, the stabilization of the resulting micro‐/nanoparticles should be carefully controlled, since the cross‐linking reaction can lead to high polydispersity.^181^ The utilization of cross‐linking agents (PEG–dialdehyde) for stabilization of the particle carrier was initially reported by Berthold et al. in 1996.^182^ Since then, such procedure is widely used in preparation of polysaccharide‐based drug carriers, especially the chitosan micro‐/nanoparticles. For example, Mao et al. developed chitosan‐based nanocarriers (ranging from 100 to 250 nm) for in vitro and in vivo gene delivery.^183^ In this approach, the chitosan–DNA complex was formed via electrostatic interaction, and sodium sulfate was facilitated as a desolvating reagent to separate nanoparticles from the solution. Glutaraldehyde was introduced for stabilizing the chitosan–DNA nanoparticles without damage of DNA.^183^ The resulting nanoparticles were further conjugated by PEG and transferrin to reduce the aggregation and enhance the transfection efficiency, respectively. Agnihotri and Aminabhavi synthesized timolol maleate‐encapsulated chitosan nanoparticles for ophthalmic delivery.^184^ The chitosan nanoparticles were formed via desolvation with the dropwise addition of acetone in the aqueous acetic acid solution containing the mixture of chitosan and timolol maleate, followed by cross‐linking with glutaraldehyde.^184^ The resulting nanoparticles had sizes ranging from 118 to 203 nm, and the drug release rate was dependent on the level of cross‐linking and the molecular weight of chitosan. Al‐Ghananeem et al. prepared hyaluronan nanoparticles for intratumoral delivery of paclitaxel.^185^ Nanoparticles were obtained from the desolvation of HA in a Tween 20 aqueous solution using sodium sulfate as desolvating agents and cross‐linked with glutaraldehyde after paclitaxel loaded into HA coacervates. Although the desolvation method simplified the purification process, the introduction of toxic glutaraldehyde would potentially impede the in vivo application if the purification of the product did not meet regulatory requirements. Moreover, the experimental optimization is always required, since various parameters such as initial molecular weight and concentration of polysaccharide, amount of desolvating agent, agitation speed, as well as molar ratio of polysaccharide/therapeutic payload can greatly influence the resulting characteristics of the nanoparticles.

#### 3.2.3 Polyelectrolyte Complexation and Ionotropic Gelation

The use of electrostatic interactions and metal–polymer coordination between polysaccharides and counterions or polyelectrolytes has drawn considerable attention. This facile and mild approach offers several unique advantages, including a nontoxic process, reversible cross‐linking, an organic solvent‐free process, and easy scaling. The materials applied in the fabrication of polyelectrolyte nanocomplexes can be divided into two main categories: (1) Small counterions or molecules, such as divalent chloride salts (e.g., CaCl_2_, MgCl_2_, CuCl_2_), pyrophosphate, citrate, sulfate; and (2) Oppositely charged macromolecules, including polyphosphates, polylysine, alkyl sulfates, polyglutamic acid, and polysaccharides.

One of the classic early studies of polyelectrolyte complexation was reported by Calvo et al.^186^ In this method, various amounts of chitosan and BSA were dissolved in aqueous solutions that contained acetic acid. Then, sodium TPP in water was subsequently mixed with chitosan solution under agitation, spontaneously producing chitosan nanoparticles. The TPP/chitosan mole ratio, stirring rate, and the degree of deacetylation of chitosan can crucially influence the particle size and surface charge. In addition, the nanoparticle size can also be affected by the molecular weight of oppositely charged cross‐linking agents, that is, employing small counterions or molecules results in smaller particle sizes than using oppositely charged macromolecules.^178^ Recently, polyion nanocomplexes based on the layer‐by‐layer deposition of sodium alginate and chitosan has been applied for improving the lipid membrane stability of nanoliposomes in the gastrointestinal tract.^187^ This study used different concentrations of chitosan and sodium alginate in aqueous solutions with pH adjusted to 5.5. The first layer was formed by addition of negatively charged nanoliposomes into chitosan solution under constant stirring for 1 h, followed by adding chitosan coated nanoliposomes into sodium alginate solution via same procedure, and resulting in the formation of alginate–chitosan coated nanoliposomes. Interactions between the ternary polysaccharide systems have been applied to develop injectable nanonetworks for controlled insulin delivery. For example, Gu et al. developed a glucose‐responsive nanoparticle‐based polymeric network^188^ that was composed of four components, including an acid‐degradable acetal‐incorporated m‐dextran, chitosan‐ and alginate‐based surface coatings, and bioactive encapsulations (i.e., glucose oxidase, GOx; catalase, CAT; and human recombinant insulin). The preparation of the nanoparticle‐based nanonetwork started by the formation of m‐dextran nanoparticles via a double emulsion (water‐in‐oil‐in‐water)‐based solvent evaporation/extraction method.^188^ A certain amount of m‐dextran in dichloromethane (DCM) was emulsified with an aqueous mixture of insulin, GOx, and CAT in specific ratios by sonication. The obtained primary emulsion was added into the chitosan and alginate aqueous solution with sustained sonication separately. The double emulsion was then transferred into chitosan and alginate aqueous solution and eliminated the DCM through agitation, followed by centrifugation. The nanonetwork was then prepared through polyelectrolyte complexation by mixing the aqueous solution of chitosan‐ and alginate‐coated nanoparticles together under constant stirring and was collected by centrifugation.^188^

In the ionotropic gelation technique, polysaccharide‐based polyelectrolytes can be used, such as the widely investigated alginate and chitosan, which can chelate with counterions to induce the gelation and form a particulate or meshwork structure. Alginate is one of the most well‐known examples and has been extensively reported. In the case of the formation of calcium alginate hydrogels, three general approaches can be used. One is the diffusion or external gelation method, where alginate solution is added dropwise into a bath of calcium chloride solution. The hydrogel matrix is formed through the diffusion of the calcium cations from the external continuous phase into the interior structure of alginate droplets.^40^, ^65^, ^189^, ^190^ The second method is the in situ gelation or so‐called internal gelation. In this approach, the insoluble calcium source (e.g., calcium salt) is mixed with an alginate solution, and the release of the calcium ions is triggered by altering the pH of the system or by increasing the solubility of calcium source, which subsequently leads to the formation of the Ca–alginate gel.^65^ The third method is the hot‐made preparation through the controlled cooling from high‐temperature hydration of a medium that consists of alginate, salt, and a sequestrant.^191^

Comparing these methods, the diffusion method is a rapid and high yield gelation process that produces an inhomogeneous Ca–alginate gel, in which the concentration of Ca–alginate gelation is dependent on the thickness of the gel.^192^ While in situ gelation provides a homogeneous ionotropic gel with a uniform distribution of calcium ions,^193^ the hot‐made preparation of the Ca–alginate gel is plainly limited to the incompatible use of heat‐labile substances. Alginate ionotropic gels prepared by different methods can exhibit distinct properties (e.g., stiffness, strength, permeability, pore size). Externally cross‐linked alginate matrix usually possesses greater matrix strength than internally alginate cross‐linked matrix, despite matrix strength can be balanced between two types of alginate matrix by adjusting the amount of cross‐linker used. Matrix flexibility can also be altered by controlling the amount and size of CaCO_3_ used in internal gelation method, but little impact on the strength of matrix. Both approaches are potentially applicable as a coating or delivery system.^189^ High molecular weights of alginate and the presence of nongelling ions can improve the uniformity of the Ca–alginate gel created with the diffusion method.^193^

#### 3.2.4 Self‐Assembly Method

Self‐assembly is a method that involves the self‐ruling organization of polysaccharide compounds into nanostructures without human interference. The joint use of self‐assembly and other methods is commonly applied for the preparation of novel supramolecular assemblies in drug delivery applications. CDs are the most widely used cyclic oligosaccharides in the drug delivery field to enhance the solubility, stability, and bioavailability of drugs. As previously mentioned, there are mainly three types of inclusion‐complex formations between substrate (drug) and CD (host), and several techniques have been used to prepare CD‐based inclusion complexes, such as the coprecipitation technique, the kneading technique, the neutralization precipitation technique, the coevaporation technique, and the microwave irradiation technique.^119^, ^194^ In the coprecipitation technique, CD is initially dissolved in an aqueous solution, and the substrate is introduced when stirring the CD solution. The solubility of CD can be increased up to 20% with elevated temperature if the substrate molecule is thermally unstable at higher temperatures. The precipitate of inclusion complexes is formed during the continuous cooling and agitation, which is then collected by centrifugation or filtration, and may be washed with a water‐miscible solvent.^195^ However, this technique is limited in its scaling‐up production ability due to the large amount of water that is required for poor solubility of CDs, as well as the massive amount of energy used for heating and cooling. Besides, some organic additives can influence the complexation efficiency of the substrate (drugs),^194^, ^195^ which is needed to take into account a particular case. The kneading technique is one of the widely used methods for inclusion complexation.^119^ In the course of its preparation, the CD is mixed with a specific amount of water or hydroalcoholic solutions to form a paste. The substrate is subsequently added to the paste and homogenized for a certain amount of time, which is then dried by vacuum desiccators.^194^ The kneading method was successfully utilized for encapsulation of various drugs in both small‐ and large‐scale production, including azomethine, sulfamethoxazole, linalool, and difluorinatedcurcumin.^196^ Neutralization precipitation is a technique used for the precipitation of ionizable inclusion complexes, which are prepared by dissolving the substrate in an alkaline solution and mixing with an aqueous CD solution. The pH of the resultant mixture is neutralized by adding a hydrochloric acid solution while stirring; then, the precipitate is formed and collected by filtration, followed by desiccation.^197^ However, this method is limited to encapsulate acid‐ and alkaline‐labile substrates.^194^ The coevaporation technique is a simple and economic method that involves the mixing of two different miscible solutions (for instance, an aqueous CD solution and an alcoholic solution of a substrate) to form an emulsion of inclusion complexes. Then, the solvent is evaporated and dried under vacuum to obtain the pulverized product.^197^ Microwave irradiation is an effective and convenient technique for the rapid complexation of CD and a substrate. In this process, the CD and substrate are dissolved in a solvent and reacted for a short period of time using a microwave oven. When the reaction is completed, the free substrate, cyclodextrin, and residual are removed by a solvent mixture, and the resultant precipitate is dried in a vacuum oven.^197^, ^198^

#### 3.2.5 Microfluidic Methods

To ensure that the sizes of the nanoparticles are evenly distributed, a homogenizer is often used in the emulsion process to reduce the sizes of the droplets in liquid–liquid dispersions, generating stable homogenized particles. However, the inherent random process makes it a nonideal strategy to fabricate polysaccharide nanoparticles in industry. Microfluidics has shown unparalleled advantages for the synthesis of polymer particles and have been utilized to produce hydrogel particles with a well‐defined size, shape, and morphology. Most importantly, during the encapsulation process, microfluidics can control the number of cells per particle and the overall encapsulation efficiency. Therefore, microfluidics is becoming a powerful approach for cell microencapsulation and the construction of cell‐based drug delivery systems.^199^

An example of Ca^2+^‐cross‐linked alginate microspheres were generated from a microfluidic device by Chen et al. They reported a versatile method of droplet microfluidics to fabricate alginate microspheres while simultaneously immobilizing an anti‐*Mycobacterium tuberculosis* complex Immunoglobulin Y (IgY) and anti‐*Escherichia coli* IgG antibodies primarily on the porous alginate carriers for specific binding and binding affinity tests.^200^ They actually presented the shape and surface structure of calcium‐cross‐linked alginate microspheres under microscopy. They were generally round with an undulating membrane. Tiny porous structures were shown in zoomed in pictures of microsphere surfaces.

**Figure 10:**
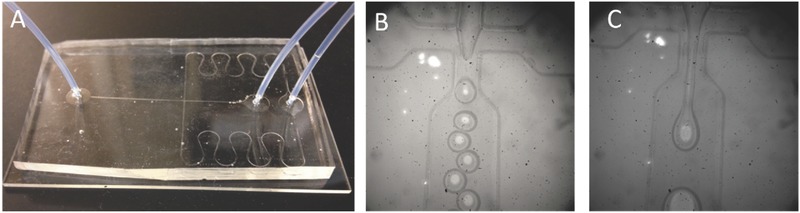
A) Photograph of microfluidic device. B,C) Microscopic photographs of alginate/oil droplets pumped out T‐junction inside the microfluidic device. Reproduced with permission.^207^ Copyright 2015.

Microfluidic devices utilize the science of manipulating and controlling fluids and particles at micrometer or sub‐micrometer dimensions to exploit a wide range of biological applications such as high‐throughput drug screening of single cell or molecular analysis and manipulation, drug delivery and advanced therapeutics, biosensing, and point of care diagnostics, among others.^201^ Fluid flow in microchannels is diffusion‐based laminar flow due to the low Reynolds numbers.^202^ Several materials have been casted to make microfluidic devices, including polymer (including polydimethylsiloxane, polymethylmethacrylate, polycarbonate, cyclic olefin copolymer),^203^ silicon,^204^ and metal.^205^ Typically, syringe pumps or microfabricated pumps provide pressure‐driven flow in the microchannels, and electrokinetic devices provide other choices for pumping liquids. Reagent solutions are manipulated inside microfluidic devices. A T‐junction type of channels is usually designed to generate droplets alternatively and fuse tow reagent droplets in a tapered chamber. In the long switch back channel, particles with nano‐ or microsizes can then be synthesized in each droplet reactor and collected at the end of device.^206^ In our group, we have designed a microfluidic‐flow‐focusing device which is consistently reproducible, readily characterized, and easy to test and use to produce homogeneous alginate microparticles (**Figure** **10**). Microparticles with the same size were pumped out of the T‐junction and then collected at 1 m CaCl_2_ solution. High speed camera recording helped to identify the process of formation of a single droplet in the microfluidic devices.^207^ Microfluidic devices allow researchers to control the physical conditions and behavior of fluids in a micro‐/nanoscaled domain to fabricate polysaccharide biomaterials, offering versatile solutions for fabrication, manufacturing, and research in the fields of cell biology, pharmacology, and tissue engineering. We believe the continued enhancements of technology of microfluidic devices will produce much smaller and uniform polysaccharide nanoparticles while maintaining portable and cheap solutions for large‐scale industrial manufacturing applications.

## 4 Preclinical Advancements

The goal of biomaterials is to assist the body's self‐healing process with the engagement of different cells/tissues as well as drug molecules. Drug delivery systems are tailor‐designed to promote the therapeutic efficacy of existing drug molecules in controlled manner. Our discussion focuses on two major categories of biomedical applications: (1) tissue engineering with regenerative medicine and (2) targeted delivery and theranostic applications in the field of treatment of diseases.

### 4.1 Tissue Engineering and Regenerative Medicine

Polysaccharides are able to form hydrogels and micro‐/nanoparticles after certain reactions, which can encapsulate drug for therapeutic application. Tissue engineering is an emerging biomedical field that aims to assist and enhance the regeneration of body tissue defects that are too large to self‐repair or to substitute for the biological functions of damages/injured organs. To promote tissue regeneration or wound healing, many protein growth factors are required. For example, some growth factors are able to induce angiogenesis, which then supplies oxygen and nutrients to cells transplanted for organ substitution to maintain their biological functions.^208^ Some growth factors are also shown to stimulate the proliferation and differentiation activity of stem cells via certain cellular signal pathways.^209^ However, the biological effects of growth factors cannot always be expected because of their poor in vivo stability, unless a drug delivery system is contrived.^208^ Various growth factors have shown to affect the proliferation and survival of multipotential stromal cells, including transforming growth factor beta (TGF‐β), the fibroblast growth factor (FGF), the VEGF, the platelet‐derived growth factor, the epidermal growth factor, the hepatocyte growth factor, and the Wnt family.^210^ Almubarak et al. summarized the role of commonly used growth factors in angiogenesis and osteogenesis and highlighted the current status of preclinical and clinical trials.^211^

**Figure 11:**
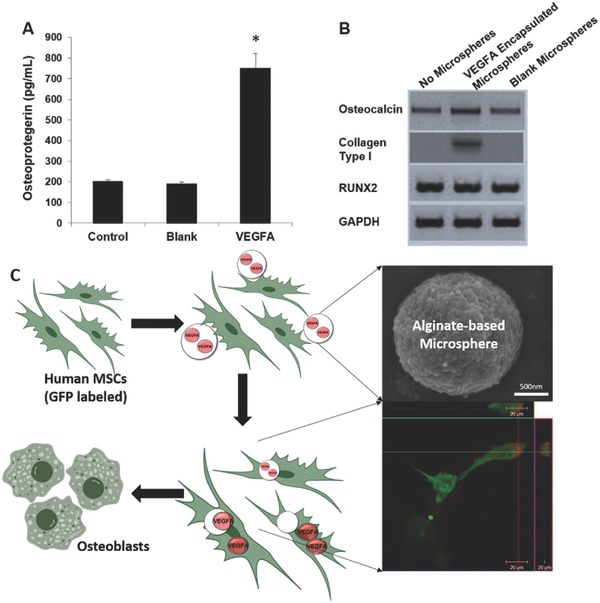
A) Protein results of human MSCs after 14 d in culture with differentiation growth medium. Sample aliquots were collected and osteoprotegerin concentration (pg mL^−1^) in the supernatant medium was measured using an enzyme‐linked immunosrobent assay (ELISA) assay (*n* = 3). * indicates significantly higher result, *p* < 0.0001. B) Reverse transcription polymerase chain reaction (RT‐PCR) results verify production of osteocalcin and RUNX2 in MSCs after 14 d in culture with osteogenic differentiation growth medium. Collagen type I was upregulated for MSCs pretreated with VEGFA‐encapsulated Alg–*g*–PEG microspheres prior to differentiation. Glyceraldehyde‐3‐Phosphate Dehydrogenase (GADPH) was used as an internal control. C) Schematic representation of hMSCs endocytosis alginate‐based microparticles (loading with VEGFA), inducing osteogenesis differentiation. Reproduced with permission.^22^ Copyright 2014, Elsevier.

Our group has reported using alginate microparticles to stealth‐deliver VEGF intracellularly to mesenchymal stem cells (hMSCs), inducing osteogenesis differentiation of hMSCs. The alginate microparticle prevented the delivered VEGF to interact with the VEGF surface receptor (VEGFR), which could potentially direct hMSCs into the osteoblast linage rather than adipocytes.^22^ Liu et al. showed that the stealth delivery of VEGF effectively contributed in the differentiation signal pathway; they found that the intracellular expression of VEGFA but not external application of the growth factor could cure osteoporosis.^212^ As expected, hMSCs could endocytose VEGFA–microparticles within 48 h coculturing and differentiate into osteoblast after 14 d (**Figure** **11**). The utilization of alginate microparticles provides a possible solution to activate the intracrine mechanism, which may be different from the paracrine mechanism with respect to directing cell fates. **Table** **1** lists examples of using different polysaccharides to control delivery of certain bioactive agents for various applications.

**Table 1:** Examples of different polysaccharides cargo system to control delivery certain bioactive agents for various applications

Polysaccharide type	Bioactive agents	Application	Role in tissue engineering	Ref.
Alginate	Amidated pectin hosting doxycycline (Antibiotics)	Wound healing	Inhibit bacterial‐infection‐caused necrosis in wound healing process	256
Alginate	VEGF	Osteoporosis	Promote osteogenesis differentiation of hMSCs rather than adipogenesis	22
Alginate	Human fibroblast growth factor 1 (FGF‐1)	Cartilage defects	Promote the in vitro development of mature adipocytes	257
	Human bone morphogenetic protein 4 (BMP‐4)			
Alginate	Human fibroblast growth factor 1 (FGF‐2)	Peripheral artery disease and coronary artery disease	Promote neovascularization and restore blood flow and tissue function of heart muscle	258
Alginate	FGF‐1	Hypoxia	Enhancement of graft neovascularization in a retrievable rat tomentum pouch	259
Alginate	Transforming growth factor‐beta (TGF‐β)	Articular cartilage defects	Controlled delivery of TGF‐β selectively to the injury site and improve the repair of articular cartilage defects in rabbit model	260
Alginate	Insulin‐like growth factor‐1 (IGF‐1)	Nervous system disorders such as stroke	Enhanced the proliferation of the encapsulated NSCs	261
Hyaluronic acid	None	Atherosclerosis	Reach the atherosclerotic lesion after systemic administration with high potential as carrier for diagnosis and therapy of atherosclerosis	261
Hyaluronic acid	VEGF	Wound healing	Promote angiogenesis and accelerate healing	262
Chitosan				
Hyaluronic acid	VEGF	Development of vascular network during implantation	Promote angiogenesis	263
Chitosan	PGDF–BB			
Poly(l‐lactide‐*co*‐glycolide) (PLGA)‐grafted hyaluronic acid	Bone morphogenetic protein‐2 (BMP‐2) and IGF‐1	Bone regeneration	Promote the attachment, proliferation, spreading, and alkaline phosphatase (ALP) activity of human adipose‐derived stem cells (hADSCs)	264
Alginate microspheres within hyaluronic acid hydrogels	TGF‐β3	Cartilage repair	Promote neo cartilage formation	265
	Parathyroid hormone related protein (PTHrP)			
Hyaluronic acid/chitosan nanoparticles embedded in porous chitosan scaffold	DNA encoding TGF‐β1	Cartilage tissue engineering	Promotion of chondrocyte proliferation	266
Glycidyl methacrylated dextran	BMP‐2	Wound healing	Periodontium drug delivery	267
Acetalated dextran	Hepatocyte growth factor fragment	Myocardial infarction (MI)	Largest arterioles, fewest apoptotic cardiomyocytes bordering the infarct, and the smallest infarcts	268
Methacrylated dextran	VEGF	Ischemia	Increase blood perfusion and angiogenesis	269
Chitosan–polyethylenimine	BMP‐2 gene	Repair of bone defect	Affect cell differentiation through a BMP‐2 signal pathway and promote new bone formation at the defect area	270

Polysaccharide‐based micro‐/nanoparticles provide protection to the protein‐based growth factors, offering a versatile release profile in a controlled manner while reducing the risk of having site effects, and are able to deliver the bioactive agent to target cells. In addition, stimulation can form different types of protein cargos, and the cellular signaling will then influence the cellular process, such as attachment, proliferation, migration, and differentiation, demonstrating the potential for applying these strategies for promoting tissue regeneration.^213^

### 4.2 Targeted Delivery and Theranostic Applications

Cancer remains one of the worlds' major causes of death^214^ and the improvement of effective therapies continues to challenge researchers. The great biocompatibility and the availability of multifunctional conjugation make polysaccharide nanoparticles arguably one of the best drug delivery vehicles for cancer treatment. With optimal size and surface properties, polysaccharide nanoparticles can be designed and engineered to increase the bloodstream circulation time and reach the target tumor lesion. Due to the enhanced permeability and retention effect to the ligands conjugation, nanoparticles are accumulated in the tumor tissue while delivery anticancer therapeutics are entrapped inside the particles, providing a higher targeting efficacy compared to traditional drug delivery methods. Conjugation target moieties also facilitate the precise delivery of chemotherapeutics, resulting in higher treatment efficiency and lower side effects.^215^ **Table** **2** lists some selected examples of polysaccharide‐based drug delivery systems encapsulated with therapeutic/diagnostic agents for cancer therapy. Although different types of polysaccharides have been assessed to develop a suitable anticancer/theranostic nanosystems (Table 2), only negligible amount of development can reach clinical trials. It should be noted that complex chemical conjugation may result in unexpected toxicity after systemic administration because of impurity of products. The future study of polysaccharide‐based CRS is seen to be centered on the improvement of sensitivity and specificity of stimuli‐responsive triggers, as well as safety profile after systemic delivery.

**Table 2:** Examples of polysaccharide‐based drug delivery systems for controlled delivery anticancer agents

Polysaccharide type	Anticancer agents	Imaging agents	Cancer type	Result and application	Ref.
Hyaluronic acid (HA)	None	Cy5.5	Xenograft subcutaneous dorsa of athymic nude mice	To visualize the biodistribution of HA nanoparticles accumulating into the tumor with a combination of passible and active targeting mechanism	271
Liposome–protamine–hyaluronic acid	TGF‐β siRNA	None	Melanoma	Induction of antigen‐specific immune response and target modification of tumor microenvironment; powerful tool for immunotherapy	272
Chitosan	siRNA for VEGRA, VEGFR1, and VEGFR2	None	Breast cancer	Suppressive effect on VEGF expression and tumor volume	273
Chitosan/alginate	Doxorubicin	None	HepG2 hepatoma cells xenografts	Induce the apoptosis of HepG2 tumor cells both in vitro and in vivo	274
Alginate	Doxorubicin	None	Liver tumor	Tumor necrosis; heart cells and healthy liver cells surrounding the tumor were not affected	275
Glycyrrhetinic acid‐modified alginate	Doxorubicin	None	Hepatoma carcinoma	Tumor inhabitation rate reach 79.3%	282
Alginate–*g*–poly(*N*‐isopropylacrylamide) (PNIPAAm)	Doxorubicin	FCR‐675	Squamous cell carcinoma	DOX‐loaded alginate–*g*–PNIPAAm micelles showed excellent anticancer therapeutic efficacy in a mouse model without any significant side effects	277
Alginate	Cisplatin(CDDP)	Cy5.5	Human caucasian ovary adenocarcinoma	Enhance delivery of CDDP into ovarian tumor tissues and improved the antitumor efficacy of CDDP, while reducing nephrotoxicity and body weight loss in mice	278
*N*‐trimethyl chitosan	Cisplatin–alginate complex	None	Human ovarian and lung carcinoma	Induce apoptosis	279
Hyaluronic acid	Cisplatin	None	Human malignant gliomas	Induce apoptosis	280
Chitosan	Destran–doxorubicin	None	Various cancer types	Induce apoptosis and shrink tumor size	281
Hyaluronic acid	Cisplatin siRNA that downregulate antiapopotic genes overexpressed in cisplatin resistant tumor	Indocyanine green	Lung cancer	Overcome the Multidrug resistance effect of lung cancer in xenograft model and induce apopotosis	282

**Figure 12:**
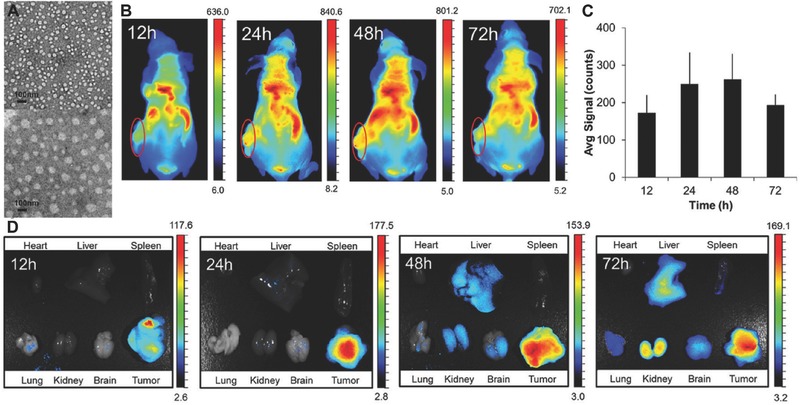
A) Transmission electron microscopy (TEM) images of CSO—ss—SA (up) and PTX loaded CSO—ss—SA) (down). B) In vivo whole body images and C) average fluorescent signal. D) Fluorescent images of organs of tumor bearing nude mice after injection DiR‐labeled CSO—ss—SA for 12, 24, 48, and 72 h. Reproduced with permission.^54^ Copyright 2015, Elsevier.

One attractive strategy for intracellular controlled release of anticancer agents is the exploitation of the redox‐responsive system, which contains disulfide bonds that can be cleaved by overexpressed glutathione in tumor cells. For example, Hu et al. reported chitosan‐based glycolipid‐like CSO—ss—SA (CSO: chitosan; SA: stearylamine) micelles for selective release of DOX/PTX by responding to the reducing environment in tumor cells.^54^ CSO—ss—SA micelles exhibited a desired reduction‐sensitivity as they were able to promote fast degradation and release of the drug in 10 × 10^−3^ m of GSH. An in vitro drug release study indicated that CSO—ss—SAs could quickly deliver the drug into the human ovarian cancer cells (SKOV‐3) and human normal liver cells (L‐02) through endocytosis pathway with significant higher delivery efficiency in SKOV‐3 compared to L‐02. Besides, the cellular inhibition rate of PTX‐loaded CSO—ss—SA micelles was positively correlated with the intracellular GSH concentration in SKOV‐3 cells. A mouse xenograft model study showed that CSO—ss—SAs could effectively deliver the drug into tumor tissue via the EPR effect (**Figure** **12**). Although there was high deposition of CSO—ss—SAs in the liver and spleen, the drug release mainly existed in the tumor. Compared with Taxol at the same doses, PTX‐loaded CSO—ss—SA micelles provided a distinguished antitumor effect with a rather low dose of PTX. Overall, this study emphasizes that the rational design of a selective redox‐responsive system could serve as a smart platform for drug delivery with the least toxicity and rapid intracellular drug liberation in tumor cells.

**Figure 13:**
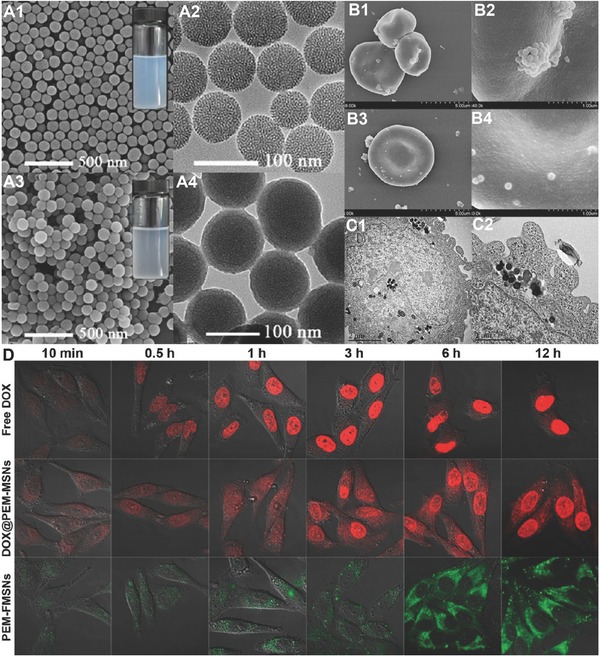
A1,A3) Scanning electron microscopy (SEM) and A2,A4) TEM images of (A1/A2) MSN and (A3/A4) PEM–MSNs with adjacent photographs showing good dispersity in water. SEM images of human red blood cells (RBCs) cultured on B1,B2) MSNs and B3,B4) PEM–MSNs. C) TEM images of intracellular uptake of PEM–MSNs in HeLa Cells; (C2) was the zoomed in image of (C1). D) Confocal laser scanning microscopy (CLSM) images of HeLa cell coincubation with free DOX, DOX encapsulated PEM–MSNs, and PEM–FMSNs for 10 min, 0.5 h, 1 h, 3 h, 6 h, and 12 h. Reproduced with permission.^216^ Copyright 2014, ACS.

In addition to redox‐triggered drug release, the pH difference between tumor and normal tissues, as well as between the cytoplasm and endosomes can also be harnessed for controlled release of the chemotherapeutics. For instance, Feng et al. described the surface coating of DOX‐loaded mesoporous silica nanoparticles (MSNs) with multilayers of alginate/chitosan to impart pH responsiveness of the nanocarriers (DOX@PEM–MSNs; i.e., DOX‐loaded polyelectrolyte multilayer (PEM)–green fluorescence FITC‐labeled MSNs (FMSNs)).^216^ The release of DOX was triggered by acidic intracellular or extracellular environments. An in vitro study on HeLa cells showed that the intracellular release of DOX from nanocarriers was pH dependent (lowering pH increased the release rate), and a sustained DOX accumulation in the nucleus led to prolonged therapeutic efficacy (**Figure** **13**). Moreover, an in vivo evaluation in healthy rats demonstrated that these DOX@PEM–MSNs carriers exhibited longer systemic circulation time and slower plasma elimination rate than free DOX. Compared with unmodified MSNs, the PEM–FMSNs showed superior hemocompatibility in terms of low hemolytic and cytotoxic effects against human red blood cells (RBCs), which endorses them as potential candidates for systemic delivery.

Dysregulated enzyme expression is often associated with numerous diseases, particularly cancer, inflammatory, and infectious diseases. Certain upregulated enzymes (e.g., matrix metalloproteinases, cathepsins, caspases, thrombins, glucuronidase) could be considered as specific endogenous triggers for the release of therapeutic and diagnostic agents. HA‐coated MSNs loaded with DOX were also reported by Zhang et al. They grafted the biotin–HA on desthiobiotin decorated MSNs via a streptavidin‐mediated cross‐linkage, which prevented the DOX release from the pores of the MSNs.^217^ Once the MSN–HA/DOX was specifically taken up by CD44‐positive cancer cells by receptor‐mediated endocytosis, DOX was released from the pore of MSN by competitive binding of cytoplasmic biotin and desthiobiotin to streptavidin. In vitro examinations showed that MSN–HA could be internalized by HT‐29 and Colon‐26 cells (CD44 positive), and the release of DOX was promoted significantly in the presence of hyaluronidase (HAase) and/or biotin. HA‐coated MSNs displayed higher cell viability than bare MSNs. An in vivo safety evaluation demonstrated that despite that MSN–HA showed little nonspecific interaction with proteins, blood cells, and macrophages, MSN–HA could significantly improve the biocompatibility of MSNs by surface coating HA. Evaluation of MSN–HA/DOX on a colon‐26 xenograft tumor model showed that MSN–HA/DOX had better antitumor effect than free DOX, owing to the presence of extracellular matrix‐localized HAase and intracellular biotin in the tumor site that triggered the disintegration of biotin–HA from MSNs, which thus enhanced its antiproliferative activity in a solid tumor.

Zhang et al. reported using glycyrrhetinic acid (GA)‐modified alginate nanoparticle to target delivery of DOX to kunming mice for curing liver cancer. GA is a commonly used bioactive ligand for modification of DDS and results in additional accumulation of drug molecules in the liver sites. Passive targeting with enhanced permeability was also a leading cause for liver cancer accumulation. Instead of focusing on the therapeutic effect of shrinking the tumor, the authors also evaluated the side effects with regards to the DOX chemotherapy. The in vivo study results suggested that after a single tail‐vein injection of 7 mg kg^−1^ body weight, the concentration of DOX in the liver reached 67.8 ± 4.9 µg g^−1^, which was 2.8‐fold and 4.7‐fold higher compared to non‐GA modified alginate nanoparticles and free DOX HCl. A histological examination showed tumor necrosis in both experimental groups. Most importantly, the heart cells and the liver cells surrounding the tumor were not affected by administration of DOX/GA–ALG NPs, whereas myocardial necrosis and apparent liver cell swelling were observed after DOX·HCl administration.^217^

The RNAi technique has open a new route for cancer therapy and several candidates are being clinically tested. In the development of RNAi‐based techniques, imaging methods provide a visible and quantitative solution to investigate the therapeutic effect at anatomical, cellular, and molecular levels and they are able to noninvasively trace the distribution and study the biological processes in preclinical and clinical stages.^218^ Nanocarrier‐mediated delivery of RNAi therapeutics usually encounters different biological barriers, including reaching the circulation, crossing the vascular barrier, cellular uptake, and endosomal escape. With advancements in chemical modification and nanotechnology, polysaccharide nanoparticles are diverse in size and charge and are widely applied as platforms for simultaneous gene/drug delivery and imaging.^14^ Yoon et al. reported a novel type of biodegradable hyaluronic acid‐*graft*‐poly(dimethylaminoethyl methacrylate) (HPD) conjugates that can form complexes with siRNA and that can be chemically cross‐linked via the formation of the disulfide bonds under facile conditions to exhibit high stability in 5% serum solution over the un‐cross‐linked ones. The in vivo study, which was performed using FPR675‐labeled HPD with siRNA complexes, showed the efficacy of selective accumulation of the complexes at the tumor site after intravenous injection into tumor‐bearing mice, achieving a successful gene silencing effect while being able to be monitored with a whole‐body near infrared flurescence (NIRF) imager.^219^ While the application of polysaccharide coated particles show promising result in research, there are still obstacles before more clinical trials are tested. One problem is to target sites that are located farther from the magnetic source. Future research should focus on designing multimodality imaging probes with polysaccharide coatings to enhance the use of particle‐based imaging‐based contrasts, offering versatile solutions for early cancer detection and monitoring.^220^

**Figure 14:**
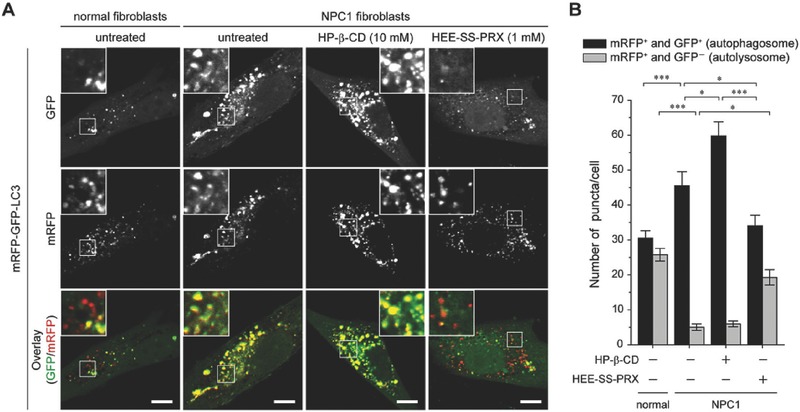
A) CLSM images of normal and NPC1 fibroblast cells expression mRFP‐GFP‐LC3 coincubated with HP–β‐CD and HEE—ss—PRX nanoparticles at the concentration of 10 × 10^−3^ and 1 × 10^−3^ m. B) Quantification of cell population with production of autophagosomes (mRFP^+^ and GRP^+^) and autolysosome (mRFP^+^ and GRP^−^). Reproduced with permission.^221^ Copyright 2015, American Society for Biochemistry and Molecular Biology.

Aside from cancer therapeutics, CD‐based supramolecular assemblies have received attentions in confronting genetic and rare diseases.^148^, ^221^ A series of stimuli‐cleavable β‐CD‐based polyrotaxanes (PRXs) have been investigated by Tamura and Yui.^222^ According to a recent study, a redox‐responsive β‐CD‐threaded PRX has been developed for the treatment of NPC disease,^221^ which was achieved by the controlled released of β‐CDs from HEE group‐modified Pluronic P123 via intracellular disulfide bonds cleavage. In this report, they compared the efficacy of PRXs and hydroxypropyl‐β‐CD (HP‐β‐CD) for treatment of the autophagy failure in NPC disease. Usually, an increased number of LC3‐positive puncta can be observed from NPC patient‐derived fibroblasts (NPC1 fibroblasts). When treated with HP‐β‐CD, the autophagic degradation activity was further disturbed by the increasing amount of LC3‐positive puncta and levels of p62 in the NPC1 fibroblasts, whereas the PRX‐based treatment diminished both the amount of LC3‐positive puncta and levels of p62 in the NPC1 fibroblasts via the mammalian target of rapamycin (mTOR)‐independent pathway. The evaluation of the mRFP‐GFP‐LC3 reporter gene expression that demonstrated the redox‐responsive PRXs mediated the generation of autolysosomes to approach for autophagic protein degradation (**Figure** **14**). In this regard, the developed β‐CD‐threaded bioresponsive PRXs offered a promising treatment for NPC disease based on simultaneously improving the cholesterol accumulation in lysosomes and damaged autophagy functionality in NPC1 fibroblasts.

**Figure 15:**
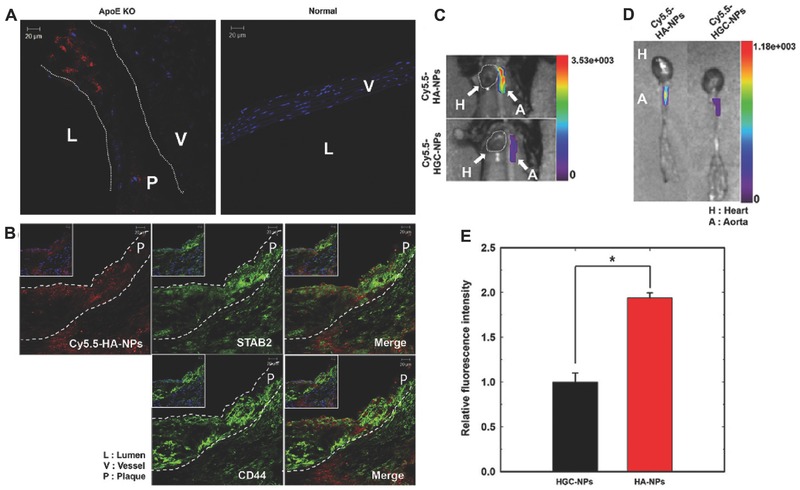
A) Fluorescent images of Cy5.5‐labeled HA‐NPs in atherosclerotic plaque in ApoE KO and normal mice. B) Zoom in fluorescent images of HA‐NPs in isolated plaques with immunostaining of Stabilin‐2 (STAB2) and CD44 with insetted images show the 4′,6‐diamidino‐2‐ phenylindole (DAPI) nuclei stain. C) In vivo live image of HA‐NP in atherosclerotic lesion in ApoE KO mice. D) Fluorescent images of isolated aorta after sacrificing the ApoE KO mice. E) Quantitatively analysis of the fluorescent intensity of HGC‐NPs and HA‐NPs form atherosclerotic lesion images. Reproduced with permission.^224^ Copyright 2015, RSC.

Natural polysaccharides also play important roles in the diagnosis and therapy of cardiovascular diseases due to their unique features including binding affinities to atherothrombotic sites, immunomodulation and therapeutic effects as well as their use as a platform for therapeutics delivery.^223^ For example, the recognition of stabilin‐2 and CD44 receptors by HA during the pathogenic process of atherosclerosis has been explored for active targeting theranostics.^224^, ^225^ Recently, Lee et al developed HA‐NPs as therapeutic carriers for active targeting atherosclerosis,^224^ which was prepared through self‐assembly of HA‐5b‐cholanic acid‐Cy5.5 conjugates. The evaluation of cellular internalization of HA‐NPs demonstrated the stabilin‐2 or CD44 receptor‐mediated endocytosis mechanism, as cellular uptake of HA‐NPs was significantly inhibited by the pretreatment of an excess amount of free HA. In vivo fluorescence imaging of atherosclerotic lesion by tail vein injection of Cy5.5‐labeled HA‐NPs into ApoE‐deficient mice revealed that 24 h postinjected HA‐NPs successfully highlighted the atherosclerotic lesion with a stronger signal than the normal aorta. The confocal microscopy imaging showed colocalization of the stabilin‐2/CD44 antibody and HA‐NPs in the atherosclerotic plaque (**Figure** **15**A,B). Besides, in vivo fluorescence imaging also demonstrated superior targeting efficiency of HA‐NPs compared to passively targeted HGC‐NPs (Figure 15C–E). Overall, the study described the potential theranostic application of HA‐based nanopolyplexes for atherosclerosis.

## 5 Clinical Translations: Progress and Challenges

Despite the great potential of polysaccharide‐based DDSs in various preclinical studies of disease treatment, they are still elusive to the market and only limited amounts of products have entered clinical trials. We have listed some ongoing and completed clinical trials for polysaccharide‐based nanoproducts that are not limited to particulate DDSs but can be used for other therapeutic applications as well (**Table** **3**). There are several types of polysaccharide products based on a drug‐conjugated delivery system, which can be modulated to be stimuli‐responsive or receptor‐mediated targeting.^13^ Five of the known polysaccharide‐based conjugates for anticancer treatment in clinical tests are AD‐70, DE‐310, Delimotecan, ONCOFID‐P‐B, and CRLX101.

**Table 3:** Examples of polysaccharide‐based DDSs in clinical trials. IVI, intravenous infusion; CDP, cationic cyclodextrin polymer; AD–PEG, adamantane polyethylene glycol; hTf, human transferrin protein

Polysaccharide	Product name	Composition	Delivery route	Diseases or conditions	Development stage	Country, year^[Ref.]^
Dextran	AD‐70	Doxorubicin, dextran	IVI	Refractory solid tumors	Phase I discontinued	Germany, 1993^226^
	DE‐310	Exatecan mesylate, carboxymethyl‐dextran	IVI	Advanced solid tumors	Phase I	Netherlands, UK, Canada, 2005^231^
	Delimotecan (MEN 4901/T‐0128)	Camptothecin (T‐2513), carboxymethyl‐dextran	IVI	Solid tumors	Phase I	Netherlands, Italy, 2008^283^
Chitosan	Milican	Holmium‐166, chitosan	Percutaneous injection	Small hepatocellular carcinoma	Phase II	South Korea, 2006^236^
Hyaluronic acid	RadiaPlex	Sodium hyaluronate	Topical skin	Radiation dermatitis	Phase III	USA, 2007^284^
	ONCOFID‐P‐B	PTX, hyaluronic acid	Intravesical instillation	Bladder cancer	Phases I, II	Italy, 2011^233^
Cyclodextrin	CALAA‐01	siRNA (RRM2), CDP, AD–PEG, hTf	IVI	Solid tumors	Phase I	USA, 2008^285^
	CRLX101/IT‐101	β‐Cyclodextrin, PEG copolymer–camptothecin	IVI	Ovarian/tubal/peritoneal cancer	Phases I, II	USA, 2012^286^
				Rectal cancer	Phases I, II	USA, 2013^287^
				Advanced solid tumors	Phase I	USA, 2015^134^, ^288^
				Lung cancer	Phases I, II	USA, 2016^289^
Alginate	DIABECELL	Neonatal porcine islets, poly‐l‐ornithine, alginate mixture	Xenotransplantation	Type I diabetes	Phases I, II, III	New Zealand, 2009^240^
	IK‐5001	Calcium gluconate, sodium alginate	Intracoronary injection	Acute/ST‐elevation myocardial infarction, congestive heart failure	Phase I	USA, Germany, Israel, 2010^241^
	OligoG CF‐5/20	Alginate oligosaccharide	Inhalation	Cystic fibrosis	Phases I, II	UK, Norway, 2014^242^

*AD‐70*: AD‐70 is a dextran–anthracycline conjugate that is consists of a oxidized dextran polymer with molecular weight of 70 000 Da and doxorubicin conjugations through the Schiff‐base reaction with glycine attached on dextran.^226^ The principle of this conjugation approach was based on the concept that hypoxic tumor milieu are expected to promote the liberation of active drugs. In phase I of the study, AD‐70 was administered via intravenous infusion in 13 patients.^226^ Dose‐limiting toxicities (DLTs) including significant thrombocytopenia and hepatotoxicity were observed in several patients; they were attributed to specific uptake of dextran–doxorubicin conjugates by MPS, since the dextran (glucose polymer) can be recognized by glucose transporters on macrophages. Besides, the Schiff‐base formation can certainly yield aldehyde residues that could induce toxicity.^227^ Despite its promising results for Schiff‐base‐mediated tumor selectivity in an animal model, the progress to the next clinical phase was discontinued. During the phase I clinical trial, AD‐70 showed unexpected toxicity due to the immunogenic effect caused by non‐biodegradable nanoformulation that consists of modified side chain of oxidized dextran.^228^

*DE‐310*: DE‐310 is a macromolecular DDS that was discovered by Daiichi Pharmaceutical Co., Ltd. It is composed of a topoisomerase I inhibitor (DX‐8951f, a camptothecin analogue), and a biodegradable carboxymethyl–dextran polyalcohol polymer that are covalently attached via a Gly–Gly–Phe–Gly peptidyl linker.^229^ The design rationale of this macromolecular carrier was intended to afford passive targeting based on the EPR effect and the controlled release of the parent drug DX‐8951f using enzymatic cleavage of the peptidyl spacer by lysosomal proteases (cathepsins).^230^ A phase I clinical trial with DE‐310 revealed that in a total of 27 patients, one patient with metastatic adenocarcinoma achieved complete remission. Another patient with metastatic pancreatic cancer achieved partial remission. And a total of 14 patients had stabilized disease progression.^231^ Neutropenia, thrombocytopenia, and hepatotoxicity were the main DLTs. The study concluded that DX‐8951 was sustainably released from DE‐310 over a prolonged period, yet there was no detectable drug concentration in red blood cells, skin, and saliva, which supportively implies that DE‐310 could improve the therapeutic index of drug DX‐8951f. However, the insufficient sample size and data prevent clear conclusions from being drawn.

*Delimotecan*: Delimotecan is another carboxymethyl–dextran conjugate containing the camptothecin analogue 10‐(3′‐amino‐propyloxy)‐7‐ethyl‐(20S)‐camptothecin (T‐2513). T‐2513 is bound to the polymer via the triglycine linker, which can be specifically cleaved by cathepsin B and subsequently release the active drug.^232^ Cathepsin B is a lysosomal cysteine protease that is upregulated in a wide variety of human tumors; hence, the presence of the triglycine linker is important for enhancing tumor selectivity and reducing toxicity. In a phase‐I study, 22 patients received the Delimotecan treatment, and two partial remissions were observed in patients with head and neck cancer. However, adverse hematological effects such as leukopenia hematologic leukocytopenia and neutropenia and nonhematologic symptoms including skin rash, fatigue, and diarrhea had occurred after Delimotecan therapy. The clinical trial confirmed that Delimotecan had a prolonged circulation half‐life and enhanced Delimotecan retention in tumor tissues (especially when the tumor is enriched in tumor‐associated macrophages) as well as the ability to increase the release of T‐2513 via enzymatic cleavage.

*ONCOFID‐P‐B*: ONCOFID‐P‐B is a PTX–HA bioconjugate supplied by Fidia Farmaceutici S.p.A. that has entered the phase‐I evaluation.^233^ The covalent conjugation between PTX and HA was intended to improve the hydrophilicity of the active pharmacophore.^234^ A total of 16 patients with bladder cancer were treated by intravesical instillation of ONCOFID‐P‐B acid solution. No DLT was observed during the treatment, and drug concentrations were always under the detectable level; in addition, 9 patients achieved complete remission. However, 50% of the total patients experienced recurrence or progression.^233^ The study results demonstrated that ONCOFID‐P‐B was safe for treatment of nonmuscle invasive bladder cancer. Moreover, HA‐based bioconjugates can certainly improve the bioavailability of active drug PTX for intravesical chemotherapy.

*CRLX101*: CRLX101 is a polysaccharide‐based compound discovered by Cerulean Pharma Inc. It consists of active pharmacophore camptothecin that covalently attached to a CDP. The rationale of this approach was to encapsulate the hydrophobic camptothecin via intra‐ and intermolecular interactions between adjacent polymer units (cyclodextrin and polyethylene glycol) and lead to self‐assembles in aqueous solution.^133^ The resulting nanoparticles varied in size from 20 to 60 nm, due to its neutral surface charge and presence of PEG blocks that together provide a stealth effect to avoid nonspecific uptake by mononuclear phagocyte system.^235^ Moreover, the physical encapsulation of camptothecin in CRLX101 nanoparticles prevents camptothecin from enzymatic degradations in circulation. Previous clinical data demonstrated that CDP‐based DDSs can address not only plasma solubility and toxicity, but also the therapeutic index of camptothecin.^132^, ^134^

*Milican*: Unlike the above‐mentioned polysaccharide conjugates, Milican consists of a radioisotope holmium‐166 (^166^Ho(NO_3_)_3_·5H_2_O), which complexed with chitosan (2‐deoxy‐2‐amino‐d‐glucose) polymer as an embolic platform for ablative radiotherapy. The phase‐IIb clinical study shows the outstanding efficacy and long‐term safety of Milican for the treatment of small hepatocellular carcinoma (<3 cm in size) through intratumoral injection with ultrasonographic guidance.^236^ However, the effectiveness for treatment of larger tumors is currently undergoing for further evaluation.

*CALAA‐01*: CALAA‐01 was a pioneering targeted siRNA nanotherapeutic that was developed by Davis in 1996.^237^ This delivery system consists of an anti‐RRM2 (ribonucleotide reductase subunit 2) siRNA payload and cyclodextrin‐containing polymer particle core attached with AD–PEG group and some AD–PEG covalently linked to human transferrin (Tf–PEG–AD) for tumor targeting and cellular internalization. The imidazole residues are also present to promote the endosomal escape, which exploit the protonation of amines in an acidic environment and induce the influx of protons and chloride ions into endosome, elevating the osmotic pressure and resulting in the disruption of endosomal membrane.^238^ The human phase Ia/Ib clinical data from 24 patients with different cancers showed that CALAA‐01 was well tolerated during the initial dose escalation.^131^ However, two patients were experiences with DLTs after the trials were reopened. Although the delivery system of CALAA‐01 has been proved effective for targeted delivery, the full ability of CALAA‐01 failed to meet its primary end point in this trial.^239^

In addition to the anticancer therapeutics, a number of polysaccharide‐based DDSs were also used for treatment of other types of diseases, such as type I diabetes (DIABECELL),^240^ heart disorders (IK‐5001),^241^ and cystic fibrosis (OligoG CF‐5/20).^242^ These examples of polysaccharide‐based nanotherapeutics undergoing clinical trials certainly played an important role in the future development of polymeric nanomedicine. However, opportunities have always been accompanied by challenges. Translational nanomedicine is a relatively new interdisciplinary field that has revolutionized the traditional knowledge of disease and therapy through cutting edge bionanotechnology.^243^ Therefore, it is challenged by limited previous experience with addressing various concerns, such as nanoparticle formulation, delivery mechanisms, toxicity investigation, and revealing the biochemical basis of the interactions between NPs and complex biological systems.^244^

First, one of the major considerations is the design and formulation of polysaccharide‐based DDSs. Natural‐based polysaccharides are not a single discrete chemical system, as they vary in number and distribution of repeating building blocks along the polymer backbone.^20^, ^245^ Molecular weight and composition are therefore important influences on the solubility, chain flexibility, intra‐ and intermolecular forces, carrier size/shape, loading capacity, and surface charge. These physicochemical properties can subsequently determine the biophysiological behavior, such as plasma solubility, aggregation states, and immunogenicity. In this regard, regulatory control including the bench‐top synthesis and characterizations must be taken into account for successful clinical translation. The dose of polysaccharide‐based products may need to be scaled up from animal models to human trials, since statistical analyses showed that only less than 1% of the injected dose can reach the desired target sites for most published researches.^246^ In this case, reproducibility is another issue for manufacturing, since the structure of polysaccharide‐based polymers varies from bench to bench and time to time in terms of the source purchased, molecular weight variation, functional group modification, and purification.^13^

Second, knowledge of the degradation profile of different polysaccharides will also be very important for the design of the polysaccharide‐based DDSs. Breaking up the noncovalent cross‐linking (intra‐ and intermolecular forces) networks is usually the initial step. For the nondegradable cross‐link, the degradation of the polysaccharide backbone will become the leading process of polysaccharide‐based DDSs in vivo.

In a biological environment, polysaccharides usually undergo enzymatic or hydrolytic degradation into nontoxic byproducts. The degradation process usually begins with the random breaking up of β‐1,4‐ glycosidic bonds followed by the observation of *N*‐acetyl linkage deacetylation degree. As the average molecular weight decreases, the degree of deacetylation increases, resulting in a polysaccharide backbone scission and destruction of side functional groups, including carbonyl, amine, and hydroxyl.^247^ It is difficult to control the polysaccharide backbone degradation in vivo and predict the clear out mechanism of polysaccharide‐based DDSs, as the incomplete degradation will result in a burst release and compromised mechanical properties. Recent studies have designed polysaccharide‐based stimuli‐responsive (pH, thermal, mechanical force) materials to control or tune the drug release profile during in vivo application.^248^ However, more animal studies should focus on monitoring the clearance and degradation of polysaccharide‐based materials in the long run.

Another challenge is to consider the heavy reliance on the EPR effect for passive targeting in oncology. In principle, nanoparticles with sizes less than 500 nm can cross the tumor blood vessels due to the irregular gaps developed by less tightly formed endothelial cells. However, the size of the gaps varies with the type and stage of tumor.^246^ Hence, the heterogeneity of the EPR effect and limited relevant experimental information from patients can certainly result in high levels of uncertainty for delivery efficiency.^249^ In this respect, development of specific biomarkers or imaging agents to determine the strong EPR effects in patients for preselecting appropriate patients could be an option.^244^, ^250^ Once the nanoparticles have reached the target sites, they have to deal with the intratumoral microenvironment to across the tumor vascular barrier. However, nanoparticles and even smaller chemotherapeutics can only insufficiently diffuse into deep tumor space. This is due to the abnormalities of the vasculature development and the rising interstitial fluid pressure inside the tumor; together, this forms a barrier for transportation of chemotherapeutics, nanoparticles, imaging agents, etc.^251^ These obstacles have been associated with drug resistance to certain chemotherapeutics.^252^ One possible solution is to revert the abnormalities and function of tumor vessels to a relatively normal state by using antiangiogenic therapy.^253^ This in turn can ultimately improve the response to therapeutic treatment and control the tumor progression.

Concerns have also expressed that the in vitro cellular study and in vivo animal model do not fully simulate the physiology and pathophysiology in humans, in particular, the xenografted human tumors in immunodeficient mice, which may not be able to mimic the true tumor microenvironment and predict therapeutic response in human patients.^135^, ^254^, ^255^ Therefore, this could be a grand challenge in estimating possible outcomes before human trials start using preclinical results. Nevertheless, genetically engineered mice models can be useful to enhance our understanding of translational nanomedicine with more realistic approaches.^254^ In summary, like other types of nanoparticle, budding success of clinical translation for polysaccharide‐based nontherapeutic medicines relies on the integration of multidisciplinary knowledge (involving life science, clinical medicine, material science, chemistry, and engineering) and collaboration among regulatory authorities, pharmaceutical companies, academics, and governments.

## 6 Concluding Remarks

Polysaccharide‐based DDSs have emerged as one of the major naturally based polymers for biomedical application due to their excellent biocompatibility and biodegradability, structural stability, broad source, and versatile chemical compositions. Various chemical modifications of chemistry have been explored to increase the functionalities of the polysaccharide polymers. Meanwhile, novel engineering techniques and devices have been developed for DDS fabrications. These have generally made it possible for encapsulating different types of drug molecules (e.g., protein, oligonucleotides, small molecules) with a desirable release profile to target tissue and great pharmacokinetic/pharmacodynamic (PK/PD) properties. The preclinical and clinical studies represent the possibility of utilizing polysaccharide‐based DDSs to enhance the therapeutic efficacy of biopharmaceutics. Despite the largely evolving knowledge and techniques, few of the polysaccharide‐DDSs have been translated into clinical studies due to limit knowledge regarding their drug release properties, targeting and therapeutic efficacy, and degradation profile. Therefore, a better understanding of material/tissue interactions is greatly needed in the field of polysaccharide‐DDSs. While compiling more convincing characterizations both in vitro and in vivo would be helpful, utilizing additional engineering modeling and monitoring techniques will also be useful for predicting the therapeutic response for clinical applications. Furthermore, the continuing development of de novo material fabrication techniques will produce better, stable, and evenly distributed polysaccharide‐based drug carriers that can be used to tailor disease targeting models. We foresee more clinical translations studies with polysaccharide‐based materials in the near future.

## Conflict of Interest

The authors declare no conflict of interest.

## References

[1] a) A. R. Kirtane , S. M. Kalscheuer , J. Panyam , Adv. Drug Delivery Rev. 2013, 65, 1731; 10.1016/j.addr.2013.09.001 PMC3849460 24036273

[2] a) T. Lammers , L. Y. Rizzo , G. Storm , F. Kiessling , Clin. Cancer Res. 2012, 18, 4889; 22829203 10.1158/1078-0432.CCR-12-1414

[3] a) H. Maeda , J. Wu , T. Sawa , Y. Matsumura , K. Hori , J. Controlled Release 2000, 65, 271; 10.1016/s0168-3659(99)00248-5 10699287

[4] Y. Matsumoto , J. W. Nichols , K. Toh , T. Nomoto , H. Cabral , Y. Miura , R. J. Christie , N. Yamada , T. Ogura , M. R. Kano , Y. Matsumura , N. Nishiyama , T. Yamasoba , Y. H. Bae , K. Kataoka , Nat. Nanotechnol. 2016, 11, 533. 26878143 10.1038/nnano.2015.342

[5] H. Maeda , K. Tsukigawa , J. Fang , Microcirculation 2016, 23, 173. 26237291 10.1111/micc.12228

[6] D. S. Pisal , M. P. Kosloski , S. V. Balu‐Iyer , J. Pharm. Sci. 2010, 99, 2557. 20049941 10.1002/jps.22054 PMC2857543

[7] G. A. Burdock , I. G. Carabin , Toxicol. Lett. 2004, 150, 3. 15068820 10.1016/j.toxlet.2003.07.004

[8] a) J. Liu , S. Willför , C. Xu , Bioact. Carbohydr. Diet. Fibre 2015, 5, 31;

[9] S. Dumitriu , Polysaccharides: Structural Diversity and Functional Versatility, CRC Press, Boca Raton, FL 2004.

[10] J. W. Lee , J. H. Park , J. R. Robinson , J. Pharm. Sci. 2000, 89, 850. 10861586 10.1002/1520-6017(200007)89:7<850::AID-JPS2>3.0.CO;2-G

[11] I. A. Sogias , A. C. Williams , V. V. Khutoryanskiy , Biomacromolecules 2008, 9, 1837. 18540644 10.1021/bm800276d

[12] R. J. Peach , D. Hollenbaugh , I. Stamenkovic , A. Aruffo , J. Cell Biol. 1993, 122, 257. 8314845 10.1083/jcb.122.1.257 PMC2119597

[13] A. Basu , K. R. Kunduru , E. Abtew , A. J. Domb , Bioconjugate Chem. 2015, 26, 1396. 10.1021/acs.bioconjchem.5b00242 26106905

[14] M. Swierczewska , H. S. Han , K. Kim , J. H. Park , S. Lee , Adv. Drug Delivery Rev. 2016, 99, 70. 10.1016/j.addr.2015.11.015 PMC4798864 26639578

[15] a) I. Cumpstey , ISRN Org. Chem. 2013, 2013, 417672; 24151557 10.1155/2013/417672 PMC3787328

[16] A. Aravamudhan , D. M. Ramos , A. A. Nada , S. G. Kumbar , in Natural and Synthetic Biomedical Polymers (Eds.: Kumbar C. T. L. S. G., Deng M.), Elsevier, Oxford 2014, p. 67.

[17] N. Lin , J. Huang , A. Dufresne , Nanoscale 2012, 4, 3274. 22565323 10.1039/c2nr30260h

[18] A. O. Tzianabos , Clin. Microbiol. Rev. 2000, 13, 523. 11023954 10.1128/cmr.13.4.523-533.2000 PMC88946

[19] N. B. Shelke , R. James , C. T. Laurencin , S. G. Kumbar , Polym. Adv. Technol. 2014, 25, 448.

[20] A. F. A. Martínez , E. Pérez , M. Benito , J. M. Teijón , M. D. Blanco , in The Delivery of Nanoparticles (Ed: Hashim D. A. A.), InTech, Croatia, European Union 2012.

[21] Z. Liu , Y. Jiao , Y. Wang , C. Zhou , Z. Zhang , Adv. Drug Delivery Rev. 2008, 60, 1650. 10.1016/j.addr.2008.09.001 18848591

[22] T. X. Miao , K. S. Rao , J. L. Spees , R. A. Oldinski , J. Controlled Release 2014, 192, 57. 10.1016/j.jconrel.2014.06.029 PMC4169734 24979209

[23] G. Sharma , A. R. Sharma , J.‐S. Nam , G. P. C. Doss , S.‐S. Lee , C. Chakraborty , J. Nanobiotechnol. 2015, 13, 74. 10.1186/s12951-015-0136-y PMC4619439 26498972

[24] D. Dheer , D. Arora , S. Jaglan , R. K. Rawal , R. Shankar , J. Drug Targeting 2017, 25, 1. 10.3109/1061186X.2016.1172589 27030377

[25] D. D. Breimer , Adv. Drug Delivery Rev. 1998, 33, 265. 10.1016/s0169-409x(98)00034-9 10837666

[26] J. Li , D. J. Mooney , Nat. Mater. Rev. 2016, 1, 16071. 10.1038/natrevmats.2016.71 PMC5898614 29657852

[27] M. E. Young , P. A. Carroad , R. L. Bell , Biotechnol. Bioeng. 1980, 22, 947.

[28] A. S. Timin , K. V. Lepik , A. R. Muslimov , D. A. Gorin , B. V. Afanasyev , G. B. Sukhorukov , Colloids Surf., B 2016, 147, 450. 10.1016/j.colsurfb.2016.08.034 27573039

[29] E. A. Phelps , N. O. Enemchukwu , V. F. Fiore , J. C. Sy , N. Murthy , T. A. Sulchek , T. H. Barker , A. J. García , Adv. Mater. 2012, 24, 64. 22174081 10.1002/adma.201103574 PMC3517145

[30] V. B. Bueno , R. Bentini , L. H. Catalani , D. F. S. Petri , Carbohydr. Polym. 2013, 92, 1091. 23399133 10.1016/j.carbpol.2012.10.062

[31] R. Shah , N. Saha , P. Saha , Prog. Biomater. 2015, 4, 123. 26566470 10.1007/s40204-015-0043-1 PMC4636533

[32] A. D. Drozdov , J. D. Christiansen , Modell. Simul. Mater. Sci. Eng. 2015, 23, 055005.

[33] Y. K. Yew , T. Y. Ng , H. Li , K. Y. Lam , Biomed. Microdevices 2007, 9, 487. 17520372 10.1007/s10544-007-9056-4

[34] A. A. Obaidat , K. Park , Biomaterials 1997, 18, 801. 9177859 10.1016/s0142-9612(96)00198-6

[35] N. Huebsch , C. J. Kearney , X. Zhao , J. Kim , C. A. Cezar , Z. Suo , D. J. Mooney , Proc. Natl. Acad. Sci. USA 2014, 111, 9762. 24961369 10.1073/pnas.1405469111 PMC4103344

[36] Y. Brudno , D. J. Mooney , J. Controlled Release 2015, 219, 8. 10.1016/j.jconrel.2015.09.011 26374941

[37] K. Y. Lee , D. J. Mooney , Prog. Polym. Sci. 2012, 37, 106. 22125349 10.1016/j.progpolymsci.2011.06.003 PMC3223967

[38] A. Mero , M. Campisi , Polymers 2014, 6, 346.

[39] J. Varshosaz , Expert Opin. Drug Delivery 2012, 9, 509. 10.1517/17425247.2012.673580 22432550

[40] S. N. Pawar , K. J. Edgar , Biomaterials 2012, 33, 3279. 22281421 10.1016/j.biomaterials.2012.01.007

[41] L. Li , R. Ni , Y. Shao , S. Mao , Carbohydr. Polym. 2014, 103, 1. 24528694 10.1016/j.carbpol.2013.12.008

[42] R. S. Singh , N. Kaur , J. F. Kennedy , Carbohydr. Polym. 2015, 123, 190. 25843851 10.1016/j.carbpol.2015.01.032

[43] L. Liu , M. L. Fishman , K. B. Hicks , Cellulose 2007, 14, 15.

[44] M. M. Abeer , M. C. Mohd Amin , C. Martin , J. Pharm. Pharmacol. 2014, 66, 1047. 24628270 10.1111/jphp.12234

[45] C. M. Paleos , Z. Sideratou , D. Tsiourvas , Bioconjugate Chem. 2017, 28, 1611. 10.1021/acs.bioconjchem.7b00186 28431209

[46] a) P. Laurienzo , Mar. Drugs 2010, 8, 2435; 20948899 10.3390/md8092435 PMC2953395

[47] M. N. V. Ravi Kumar , React. Funct. Polym. 2000, 46, 1.

[48] A. Bernkop‐Schnürch , S. Dünnhaupt , Eur. J. Pharm. Biopharm. 2012, 81, 463. 22561955 10.1016/j.ejpb.2012.04.007

[49] V. Grabovac , D. Guggi , A. Bernkop‐Schnurch , Adv. Drug Delivery Rev. 2005, 57, 1713. 10.1016/j.addr.2005.07.006 16183163

[50] K. M. Varum , M. H. Ottoy , O. Smidsrod , Carbohydr. Polym. 1994, 25, 65.

[51] a) K. A. Janes , P. Calvo , M. J. Alonso , Adv. Drug Delivery Rev. 2001, 47, 83; 10.1016/s0169-409x(00)00123-x 11251247

[52] S. Y. Park , H. J. Baik , Y. T. Oh , K. T. Oh , Y. S. Youn , E. S. Lee , Angew. Chem., Int. Ed. Engl. 2011, 50, 1644. 21308923 10.1002/anie.201006038

[53] W. Huang , Y. Wang , S. Zhang , L. Huang , D. Hua , X. Zhu , Macromolecules 2013, 46, 814.

[54] Y. W. Hu , Y. Z. Du , N. Liu , X. Liu , T. T. Meng , B. L. Cheng , J. B. He , J. You , H. Yuan , F. Q. Hu , J. Controlled Release 2015, 206, 91. 10.1016/j.jconrel.2015.03.018 25796347

[55] Y. Xu , L. Wang , Y. K. Li , C. Q. Wang , Carbohydr. Polym. 2014, 114, 27. 25263860 10.1016/j.carbpol.2014.08.003

[56] A. Garcia , C. Peniche‐Covas , B. Chico , B. K. Simpson , R. Villalonga , Macromol. Biosci. 2007, 7, 435. 17429804 10.1002/mabi.200700032

[57] Z. W. Jing , Y. Y. Jia , N. Wan , M. Luo , M. L. Huan , T. B. Kang , S. Y. Zhou , B. L. Zhang , Biomaterials 2016, 84, 276. 26851392 10.1016/j.biomaterials.2016.01.045

[58] M. Diolosà , I. Donati , G. Turco , M. Cadenaro , R. Di Lenarda , L. Breschi , S. Paoletti , Biomacromolecules 2014, 15, 4606. 25347288 10.1021/bm5014124

[59] M. Rafat , F. Li , P. Fagerholm , N. S. Lagali , M. A. Watsky , R. Munger , T. Matsuura , M. Griffith , Biomaterials 2008, 29, 3960. 18639928 10.1016/j.biomaterials.2008.06.017

[60] a) O. Smidsrød , T. Painter , Carbohydr. Res. 1973, 26, 125;

[61] A. Kikuchi , M. Kawabuchi , M. Sugihara , Y. Sakurai , T. Okano , J. Controlled Release 1997, 47, 21. 10.1016/s0168-3659(98)00141-2 10021486

[62] G. T. Grant , E. R. Morris , D.A. Rees , P. J. G. Smith , D. Thom , FEBS Lett. 1973, 32, 195.

[63] M. Spasojevic , G. A. Paredes‐Juarez , J. Vorenkamp , B. J. de Haan , A. J. Schouten , P. de Vos , PLoS One 2014, 9, e109837. 25347191 10.1371/journal.pone.0109837 PMC4209974

[64] G. Orive , S. Ponce , R. M. Hernandez , A. R. Gascon , M. Igartua , J. L. Pedraz , Biomaterials 2002, 23, 3825. 12164186 10.1016/s0142-9612(02)00118-7

[65] J. P. Paques , E. van der Linden , C. J. M. van Rijn , L. M. C. Sagis , Adv. Colloid Interface Sci. 2014, 209, 163. 24745976 10.1016/j.cis.2014.03.009

[66] J. L. Wu , C. Q. Wang , R. X. Zhuo , S. X. Cheng , Colloids Surf., B 2014, 123, 498. 10.1016/j.colsurfb.2014.09.047 25315499

[67] S. Jain , M. Amiji , Biomacromolecules 2012, 13, 1074. 22385328 10.1021/bm2017993

[68] M. D. Krebs , O. Jeon , E. Alsberg , J. Am. Chem. Soc. 2009, 131, 9204. 19530653 10.1021/ja9037615

[69] a) A. W. Chan , R. J. Neufeld , Biomaterials 2010, 31, 9040; 20739057 10.1016/j.biomaterials.2010.07.111

[70] K. Kesavan , G. Nath , J. K. Pandit , Sci. Pharm. 2010, 78, 941. 21179327 10.3797/scipharm.1004-24 PMC3007603

[71] T. X. Miao , S. L. Fenn , P. N. Charron , R. A. Oldinski , Biomacromolecules 2015, 16, 3740. 26509214 10.1021/acs.biomac.5b00940 PMC4679680

[72] a) J. A. Rowley , G. Madlambayan , D. J. Mooney , Biomaterials 1999, 20, 45; 9916770 10.1016/s0142-9612(98)00107-0

[73] S. N. Pawar , K. J. Edgar , Biomacromolecules 2011, 12, 4095. 22004188 10.1021/bm201152a

[74] K. H. Bouhadir , K. Y. Lee , E. Alsberg , K. L. Damm , K. W. Anderson , D. J. Mooney , Biotechnol. Prog. 2001, 17, 945. 11587588 10.1021/bp010070p

[75] a) L. B. Priddy , O. Chaudhuri , H. Y. Stevens , L. Krishnan , B. A. Uhrig , N. J. Willett , R. E. Guldberg , Acta Biomater. 2014, 10, 4390; 24954001 10.1016/j.actbio.2014.06.015 PMC4160396

[76] D. Maciel , P. Figueira , S. Xiao , D. Hu , X. Shi , J. Rodrigues , H. Tomas , Y. Li , Biomacromolecules 2013, 14, 3140. 23927460 10.1021/bm400768m

[77] M. Tian , X. Chen , Z. Gu , H. Li , L. Ma , X. Qi , H. Tan , C. You , Carbohydr. Polym. 2016, 144, 522. 27083844 10.1016/j.carbpol.2016.03.014

[78] A. I. Chou , S. O. Akintoye , S. B. Nicoll , Osteoarthritis Cartil. 2009, 17, 1377. 10.1016/j.joca.2009.04.012 PMC2753687 19427928

[79] O. Jeon , K. H. Bouhadir , J. M. Mansour , E. Alsberg , Biomaterials 2009, 30, 2724. 19201462 10.1016/j.biomaterials.2009.01.034

[80] O. Jeon , D. S. Alt , S. M. Ahmed , E. Alsberg , Biomaterials 2012, 33, 3503. 22336294 10.1016/j.biomaterials.2012.01.041 PMC3593072

[81] T. M. Shazly , A. B. Baker , J. R. Naber , A. Bon , K. J. Van Vliet , E. R. Edelman , J. Biomed. Mater. Res., Part A 2010, 95, 1159. 10.1002/jbm.a.32942 PMC2988649 20878989

[82] K. Meyer , J. W. Palmer , J. Biol. Chem. 1934, 107, 629.

[83] J. R. E. Fraser , T. C. Laurent , U. B. G. Laurent , J. Intern. Med. 1997, 242, 27. 9260563 10.1046/j.1365-2796.1997.00170.x

[84] L. Serra , J. Doménech , N. A. Peppas , Biomaterials 2006, 27, 5440. 16828864 10.1016/j.biomaterials.2006.06.011

[85] M. Litwiniuk , A. Krejner , M. S. Speyrer , A. R. Gauto , T. Grzela , Wounds 2016, 28, 78. 26978861

[86] A. C. Petrey , C. A. de la Motte , Front. Immunol. 2014, 5, 101. 24653726 10.3389/fimmu.2014.00101 PMC3949149

[87] a) J. Lesley , Q. He , K. Miyake , A. Hamann , R. Hyman , P. W. Kincade , J. Exp. Med. 1992, 175, 257; 1730918 10.1084/jem.175.1.257 PMC2119065

[88] a) G. W. Yip , M. Smollich , M. Gotte , Mol. Cancer Ther. 2006, 5, 2139; 16985046 10.1158/1535-7163.MCT-06-0082

[89] a) M. N. Collins , C. Birkinshaw , Carbohydr. Polym. 2013, 92, 1262; 23399155 10.1016/j.carbpol.2012.10.028

[90] G. Kogan , L. Soltes , R. Stern , P. Gemeiner , Biotechnol. Lett. 2007, 29, 17. 17091377 10.1007/s10529-006-9219-z

[91] S. Khetan , M. Guvendiren , W. R. Legant , D. M. Cohen , C. S. Chen , J. A. Burdick , Nat. Mater. 2013, 12, 458. 23524375 10.1038/nmat3586 PMC3633615

[92] D. Renier , V. Crescenzi , A. Francescangeli , *US7125860*, 2006.

[93] G. Hulsart‐Billström , P. K. Yuen , R. Marsell , J. Hilborn , S. Larsson , D. Ossipov , Biomacromolecules 2013, 14, 3055. 23947433 10.1021/bm400639e

[94] a) G. Huerta‐Angeles , D. Smejkalova , D. Chladkova , T. Ehlova , R. Buffa , V. Velebny , Carbohydr. Polym. 2011, 84, 1293;

[95] Y. Fan , P. Sahdev , L. J. Ochyl , J. J. Akerberg , J. J. Moon , J. Controlled Release 2015, 208, 121. 10.1016/j.jconrel.2015.04.010 PMC4430437 25869965

[96] W. Li , X. Yi , X. Liu , Z. Zhang , Y. Fu , T. Gong , J. Controlled Release 2016, 225, 170. 10.1016/j.jconrel.2016.01.049 26826304

[97] K. Liang , K. H. Bae , F. Lee , K. Xu , J. E. Chung , S. J. Gao , M. Kurisawa , J. Controlled Release 2016, 226, 205. 10.1016/j.jconrel.2016.02.004 26855049

[98] Y. Zhong , K. Goltsche , L. Cheng , F. Xie , F. Meng , C. Deng , Z. Zhong , R. Haag , Biomaterials 2016, 84, 250. 26851390 10.1016/j.biomaterials.2016.01.049

[99] H. S. Han , K. Y. Choi , H. Ko , J. Jeon , G. Saravanakumar , Y. D. Suh , D. S. Lee , J. H. Park , J. Controlled Release 2015, 200, 158. 10.1016/j.jconrel.2014.12.032 25550153

[100] H. S. Han , T. Thambi , K. Y. Choi , S. Son , H. Ko , M. C. Lee , D.‐G. Jo , Y. S. Chae , Y. M. Kang , J. Y. Lee , J. H. Park , Biomacromolecules 2015, 16, 447. 25565417 10.1021/bm5017755

[101] S. H. Kim , J. H. Kim , D. G. You , G. Saravanakumar , H. Y. Yoon , K. Y. Choi , T. Thambi , V. G. Deepagan , D. G. Jo , J. H. Park , Chem. Commun. 2013, 49, 10349. 10.1039/c3cc44260h 23942894

[102] Y. Zhong , J. Zhang , R. Cheng , C. Deng , F. Meng , F. Xie , Z. Zhong , J. Controlled Release 2015, 205, 144. 10.1016/j.jconrel.2015.01.012 25596560

[103] B. Chen , R. J. Miller , P. K. Dhal , J. Biomed. Nanotechnol. 2014, 10, 4. 24724495 10.1166/jbn.2014.1781

[104] S. F. Chen , L. Y. Li , C. Zhao , J. Zheng , Polymer 2010, 51, 5283.

[105] H. Rajpurohit , P. Sharma , S. Sharma , A. Bhandari , Indian J. Pharm. Sci. 2010, 72, 689. 21969739 10.4103/0250-474X.84576 PMC3178968

[106] J. Maia , R. A. Carvalho , J. F. J. Coelho , P. N. Simoes , M. H. Gil , Polymer 2011, 52, 258.

[107] W. Xu , J. Ding , C. Xiao , L. Li , X. Zhuang , X. Chen , Biomaterials 2015, 54, 72. 25907041 10.1016/j.biomaterials.2015.03.021

[108] J. Y. Zhu , Q. Lei , B. Yang , H. Z. Jia , W. X. Qiu , X. Wang , X. Zeng , R. X. Zhuo , J. Feng , X. Z. Zhang , Biomaterials 2015, 52, 281. 25818434 10.1016/j.biomaterials.2015.02.048

[109] M. J. Karpa , P. J. Duggan , G. J. Griffin , S. J. Freudigmann , Tetrahedron 1997, 53, 3669.

[110] D. Cao , J. He , J. Xu , M. Zhang , L. Zhao , G. Duan , Y. Cao , R. Zhou , P. Ni , Polym. Chem. 2016, 7, 4198.

[111] B. G. De Geest , W. Van Camp , F. E. Du Prez , S. C. De Smedt , J. Demeester , W. E. Hennink , Chem. Commun. 2008, 190. 10.1039/b714199h 18092083

[112] S. S. He , Y. W. Cong , D. F. Zhou , J. Z. Li , Z. G. Xie , X. S. Chen , X. B. Jing , Y. B. Huang , J. Mater. Chem. B 2015, 3, 8203. 10.1039/c5tb01496d 32262878

[113] H. Xiao , R. Qi , S. Liu , X. Hu , T. Duan , Y. Zheng , Y. Huang , X. Jing , Biomaterials 2011, 32, 7732. 21783244 10.1016/j.biomaterials.2011.06.072

[114] G. Crini , Chem. Rev. 2014, 114, 10940. 25247843 10.1021/cr500081p

[115] a) K. Liu , X. Jiang , P. Hunziker , Nanoscale 2016, 8, 36; 10.1039/c6nr04489a 27714108

[116] J. Zhang , P. X. Ma , Adv. Drug Delivery Rev. 2013, 65, 1215. 10.1016/j.addr.2013.05.001 PMC3885994 23673149

[117] a) R. Dong , Y. Pang , Y. Su , X. Zhu , Biomater. Sci. 2015, 3, 937; 26221932 10.1039/c4bm00448e

[118] a) A. Harada , Y. Takashima , M. Nakahata , Acc. Chem. Res. 2014, 47, 2128; 24911321 10.1021/ar500109h

[119] V. D. Nikolic , L. B. Nikolic , I. M. Savic , I. M. Savic , in Responsive Materials and Methods, John Wiley & Sons, Inc., 2013, p. 141.

[120] a) Z. Dan , H. Cao , X. He , L. Zeng , L. Zou , Q. Shen , Z. Zhang , Int. J. Pharm. 2015, 483, 63; 25639701 10.1016/j.ijpharm.2015.01.035

[121] K. Nobusawa , M. Akiyama , A. Ikeda , M. Naito , J. Mater. Chem. 2012, 22, 22610.

[122] P. R. Ashton , R. Königer , J. F. Stoddart , D. Alker , V. D. Harding , J. Org. Chem. 1996, 61, 903.

[123] N. Nayak , K. R. Gopidas , J. Mater. Chem. B 2015, 3, 3425. 10.1039/c4tb02114b 32262224

[124] M. F. Ma , S. G. Xu , P. Y. Xing , S. Y. Li , X. X. Chu , A. Y. Hao , Colloid Polym. Sci. 2015, 293, 891.

[125] S. Mishra , P. Webster , M. E. Davis , Eur. J. Cell Biol. 2004, 83, 97. 15202568 10.1078/0171-9335-00363

[126] S. H. Pun , M. E. Davis , Bioconjugate Chem. 2002, 13, 630. 10.1021/bc0155768 12009955

[127] D. W. Bartlett , M. E. Davis , Bioconjugate Chem. 2007, 18, 456. 10.1021/bc0603539 PMC2517011 17326672

[128] S. Hu‐Lieskovan , J. D. Heidel , D. W. Bartlett , M. E. Davis , T. J. Triche , Cancer Res. 2005, 65, 8984. 16204072 10.1158/0008-5472.CAN-05-0565

[129] J. D. Heidel , Z. P. Yu , J. Y. C. Liu , S. M. Rele , Y. C. Liang , R. K. Zeidan , D. J. Kornbrust , M. E. Davis , Proc. Natl. Acad. Sci. USA 2007, 104, 5715. 17379663 10.1073/pnas.0701458104 PMC1829492

[130] M. E. Davis , J. E. Zuckerman , C. H. J. Choi , D. Seligson , A. Tolcher , C. A. Alabi , Y. Yen , J. D. Heidel , A. Ribas , Nature 2010, 464, 1067. 20305636 10.1038/nature08956 PMC2855406

[131] J. E. Zuckerman , I. Gritli , A. Tolcher , J. D. Heidel , D. Lim , R. Morgan , B. Chmielowski , A. Ribas , M. E. Davis , Y. Yen , Proc. Natl. Acad. Sci. USA 2014, 111, 11449. 25049380 10.1073/pnas.1411393111 PMC4128111

[132] S. Svenson , M. Wolfgang , J. Hwang , J. Ryan , S. Eliasof , J. Controlled Release 2011, 153, 49. 10.1016/j.jconrel.2011.03.007 21406204

[133] T. Schluep , J. Hwang , I. J. Hildebrandt , J. Czernin , C. H. Choi , C. A. Alabi , B. C. Mack , M. E. Davis , Proc. Natl. Acad. Sci. USA 2009, 106, 11394. 19564622 10.1073/pnas.0905487106 PMC2703672

[134] G. J. Weiss , J. Chao , J. D. Neidhart , R. K. Ramanathan , D. Bassett , J. A. Neidhart , C. H. Choi , W. Chow , V. Chung , S. J. Forman , E. Garmey , J. Hwang , D. L. Kalinoski , M. Koczywas , J. Longmate , R. J. Melton , R. Morgan , J. Oliver , J. J. Peterkin , J. L. Ryan , T. Schluep , T. W. Synold , P. Twardowski , M. E. Davis , Y. Yen , Invest. New Drugs 2013, 31, 986. 23397498 10.1007/s10637-012-9921-8 PMC3774600

[135] A. J. Clark , D. T. Wiley , J. E. Zuckerman , P. Webster , J. Chao , J. Lin , Y. Yen , M. E. Davis , Proc. Natl. Acad. Sci. USA 2016, 113, 3850. 27001839 10.1073/pnas.1603018113 PMC4833237

[136] R. Namgung , Y. Mi Lee , J. Kim , Y. Jang , B. H. Lee , I. S. Kim , P. Sokkar , Y. M. Rhee , A. S. Hoffman , W. J. Kim , Nat. Commun. 2014, 5, 3702. 24805848 10.1038/ncomms4702

[137] E. Wajs , T. T. Nielsen , K. L. Larsen , A. Fragoso , Nano Res. 2016, 9, 2070.

[138] a) J. Li , NPG Asia Mater. 2010, 2, 112;

[139] P. Dandekar , R. Jain , M. Keil , B. Loretz , M. Koch , G. Wenz , C. M. Lehr , J. Mater. Chem. B 2015, 3, 2590. 10.1039/c4tb01821d 32262906

[140] P. Dandekar , R. Jain , M. Keil , B. Loretz , L. Muijs , M. Schneider , D. Auerbach , G. Jung , C. M. Lehr , G. Wenz , J. Controlled Release 2012, 164, 387. 10.1016/j.jconrel.2012.06.040 22789529

[141] a) C. B. Rodell , A. L. Kaminski , J. A. Burdick , Biomacromolecules 2013, 14, 4125; 24070551 10.1021/bm401280z PMC3851010

[142] V. D. Badwaik , E. Aicart , Y. A. Mondjinou , M. A. Johnson , V. D. Bowman , D. H. Thompson , Biomaterials 2016, 84, 86. 26826298 10.1016/j.biomaterials.2015.11.032 PMC4755830

[143] V. Badwaik , Y. Mondjinou , A. Kulkarni , L. Liu , A. Demoret , D. H. Thompson , Macromol. Biosci. 2016, 16, 63. 26257319 10.1002/mabi.201500220 PMC4891183

[144] Y. A. Mondjinou , L. A. McCauliff , A. Kulkarni , L. Paul , S. H. Hyun , Z. Zhang , Z. Wu , M. Wirth , J. Storch , D. H. Thompson , Biomacromolecules 2013, 14, 4189. 24180231 10.1021/bm400922a PMC4314287

[145] A. Tamura , K. Nishida , N. Yui , Sci. Technol. Adv. Mater. 2016, 17, 361. 27877888 10.1080/14686996.2016.1200948 PMC5101866

[146] a) S. Naureckiene , D. E. Sleat , H. Lackland , A. Fensom , M. T. Vanier , R. Wattiaux , M. Jadot , P. Lobel , Science 2000, 290, 2298; 11125141 10.1126/science.290.5500.2298

[147] a) Y. Tanaka , Y. Ishitsuka , Y. Yamada , Y. Kondo , T. Takeo , N. Nakagata , T. Higashi , K. Motoyama , H. Arima , M. Matsuo , K. Higaki , K. Ohno , T. Irie , Mol. Genet. Metab. Rep. 2014, 1, 19; 27896072 10.1016/j.ymgmr.2013.12.003 PMC5121301

[148] A. Tamura , N. Yui , Sci. Rep. 2014, 4, 4356. 24619155 10.1038/srep04356 PMC3950578

[149] S. K. Nitta , K. Numata , Int. J. Mol. Sci. 2013, 14, 1629. 23344060 10.3390/ijms14011629 PMC3565338

[150] A. El‐Faham , F. Albericio , Chem. Rev. 2011, 111, 6557. 21866984 10.1021/cr100048w

[151] a) P. S. Vlasov , A. A. Kiselev , N. S. Domnina , E. V. Popova , S. L. Tyuterev , Russ. J. Appl. Chem. 2009, 82, 1675;

[152] J. Berger , M. Reist , J. M. Mayer , O. Felt , N. A. Peppas , R. Gurny , Eur. J. Pharm. Biopharm. 2004, 57, 19. 14729078 10.1016/s0939-6411(03)00161-9

[153] N. Bhattarai , J. Gunn , M. Q. Zhang , Adv. Drug Delivery Rev. 2010, 62, 83. 10.1016/j.addr.2009.07.019 19799949

[154] S. Mizrahy , D. Peer , Chem. Soc. Rev. 2012, 41, 2623. 22085917 10.1039/c1cs15239d

[155] O. Smidsrod , G. Skjak‐Braek , Trends Biotechnol. 1990, 8, 71. 1366500 10.1016/0167-7799(90)90139-o

[156] D. A. Rees , E. J. Welsh , Angew. Chem., Int. Ed. 1977, 16, 214.

[157] D. A. Rees , Pure Appl. Chem. 1981, 53, 1.

[158] W. R. Gombotz , S. F. Wee , Adv. Drug Delivery Rev. 2012, 64, 194.

[159] J. Berger , M. Reist , J. M. Mayer , O. Felt , R. Gurny , Eur. J. Pharm. Biopharm. 2004, 57, 35. 14729079 10.1016/s0939-6411(03)00160-7

[160] Y. C. Luo , Q. Wang , Int. J. Biol. Macromol. 2014, 64, 353. 24360899 10.1016/j.ijbiomac.2013.12.017

[161] E. Tsuchida , K. Abe , Adv. Polym. Sci. 1982, 45, 1.

[162] M. N. V. R. Kumar , R. A. A. Muzzarelli , C. Muzzarelli , H. Sashiwa , A. J. Domb , Chem. Rev. 2004, 104, 6017. 15584695 10.1021/cr030441b

[163] A. Drogoz , L. David , C. Rochas , A. Domard , T. Delair , Langmuir 2007, 23, 10950. 17880248 10.1021/la7008545

[164] B. Sarmento , S. Martins , A. Ribeiro , F. Veiga , R. Neufeld , D. Ferreira , Int. J. Pept. Res. Ther. 2006, 12, 131.

[165] S. R. Mao , W. Sun , T. Kissel , Adv. Drug Delivery Rev. 2010, 62, 12. 10.1016/j.addr.2009.08.004 19796660

[166] M. George , T. E. Abraham , J. Controlled Release 2006, 114, 1. 10.1016/j.jconrel.2006.04.017 16828914

[167] M. Amidi , E. Mastrobattista , W. Jiskoot , W. E. Hennink , Adv. Drug Delivery Rev. 2010, 62, 59. 10.1016/j.addr.2009.11.009 19925837

[168] K. Akiyoshi , J. Sunamoto , Supramol. Sci. 1996, 3, 157.

[169] a) R. Gref , J. Rodrigues , P. Couvreur , Macromolecules 2002, 35, 9861;

[170] a) J. H. Park , G. Saravanakumar , K. Kim , I. C. Kwon , Adv. Drug Delivery Rev. 2010, 62, 28; 10.1016/j.addr.2009.10.003 19874862

[171] a) D. J. McClements , Soft Matter 2012, 8, 1719;

[172] Y. Ohya , M. Shiratani , H. Kobayashi , T. Ouchi , J. Macromol. Sci. Part A: Pure Appl. Chem. 1994, A31, 629.

[173] O. A. C. Monteiro , C. Airoldi , Int. J. Biol. Macromol. 1999, 26, 119. 10517518 10.1016/s0141-8130(99)00068-9

[174] a) B. Conti , T. Modena , I. Genta , P. Perugini , F. Pavanetto , Drug Delivery 1998, 5, 87; 19569999 10.3109/10717549809031383

[175] X. J. Song , H. Wu , S. Li , Y. F. Wang , X. J. Ma , M. Q. Tan , Biomacromolecules 2015, 16, 2080. 26075349 10.1021/acs.biomac.5b00511

[176] X. Zou , X. W. Zhao , L. Ye , Q. Wang , H. Li , J. Ind. Eng. Chem. 2015, 21, 1389.

[177] A. H. E. Machado , D. Lundberg , A. J. Ribeiro , F. J. Veiga , B. Lindman , M. G. Miguel , U. Olsson , Langmuir 2012, 28, 4131. 22296569 10.1021/la204944j

[178] A. Grenha , J. Drug Targeting 2012, 20, 291. 10.3109/1061186X.2011.654121 22296336

[179] T. Kissel , S. Maretschek , C. Packhauser , J. Schnieders , N. Seidel , in Microencapsulation: Methods and Industrial Applications, (Ed.: Benita S.), CRC Press, Boca Raton, FL 2006, p. 98.

[180] H. Ibrahim , C. Bindschaedler , E. Doelker , P. Buri , R. Gurny , Int. J. Pharm. 1992, 87, 239.

[181] P. Lai , W. Daear , R. Lobenberg , E. J. Prenner , Colloids Surf., B 2014, 118, 154. 10.1016/j.colsurfb.2014.03.017 24769392

[182] A. Berthold , K. Cremer , J. Kreuter , J. Controlled Release 1996, 39, 17.

[183] H. Q. Mao , K. Roy , V. L. Troung‐Le , K. A. Janes , K. Y. Lin , Y. Wang , J. T. August , K. W. Leong , J. Controlled Release 2001, 70, 399. 10.1016/s0168-3659(00)00361-8 11182210

[184] S. A. Agnihotri , T. M. Aminabhavi , Drug Dev. Ind. Pharm. 2007, 33, 1254. 18058322 10.1080/03639040701384942

[185] A. M. Al‐Ghananeem , A. H. Malkawi , Y. M. Muammer , J. M. Balko , E. P. Black , W. Mourad , E. Romond , AAPS PharmSciTech 2009, 10, 410. 19381833 10.1208/s12249-009-9222-5 PMC2690785

[186] P. Calvo , C. RemunanLopez , J. L. VilaJato , M. J. Alonso , J. Appl. Polym. Sci. 1997, 63, 125.

[187] W. Liu , J. Liu , W. Liu , T. Li , C. Liu , J. Agric. Food Chem. 2013, 61, 4133. 23566223 10.1021/jf305329n

[188] Z. Gu , A. A. Aimetti , Q. Wang , T. T. Dang , Y. L. Zhang , O. Veiseh , H. Cheng , R. S. Langer , D. G. Anderson , ACS Nano 2013, 7, 4194. 23638642 10.1021/nn400630x PMC4107450

[189] L. W. Chan , H. Y. Lee , P. W. S. Heng , Carbohydr. Polym. 2006, 63, 176.

[190] X. D. Liu , W. Y. Yu , Y. Zhang , W. M. Xue , W. T. Tu , Y. Xiong , X. J. Ma , Y. Chen , Q. Yuan , J. Microencapsulation 2002, 19, 775. 12569026 10.1080/0265204021000022743

[191] H. Papageorgiou , S. Kasapis , M. G. Gothard , Carbohydr. Polym. 1994, 24, 199.

[192] G. Skjakbraek , H. Grasdalen , O. Smidsrod , Carbohydr. Polym. 1989, 10, 31.

[193] G. O. Phillips , P. A. Williams , Handbook of Hydrocolloids, Elsevier, Cambridge MA 2009.

[194] S. Ain , B. Kumar , K. Pathak , Int. J. Pharm., Chem. Biol. Sci. 2015, 5, 583.

[195] E. M. M. Del Valle , Process Biochem. 2004, 39, 1033.

[196] a) K. P. Sambasevam , S. Mohamad , N. M. Sarih , N. A. Ismail , Int. J. Mol. Sci. 2013, 14, 3671; 23434664 10.3390/ijms14023671 PMC3588064

[197] R. Agrawal , V. Gupta , Int. J. Pharm. Front. Res. 2012, 2, 95.

[198] X. Wen , F. Tan , Z. Jing , Z. Liu , J. Pharm. Biomed. Anal. 2004, 34, 517. 15127807 10.1016/s0731-7085(03)00576-4

[199] J. Wan , Polymers 2012, 4, 1084.

[200] W. Chen , J. H. Kim , D. Zhang , K. H. Lee , G. A. Cangelosi , S. D. Soelberg , C. E. Furlong , J. H. Chung , A. Q. Shen , J. R. Soc., Interface 2013, 10, 20130566. 23966617 10.1098/rsif.2013.0566 PMC3785821

[201] L. Y. Yeo , H. C. Chang , P. P. Chan , J. R. Friend , Small 2011, 7, 12. 21072867 10.1002/smll.201000946

[202] M. Freemantle , Chem. Eng. News Arch. 1999, 77, 27.

[203] A. Alrifaiy , O. A. Lindahl , K. Ramser , Polymers 2012, 4, 1349.

[204] C. Iliescu , H. Taylor , M. Avram , J. Miao , S. Franssila , Biomicrofluidics 2012, 6, 016505. 10.1063/1.3689939 PMC3365353 22662101

[205] G. Li , M. Parmar , D.‐W. Lee , Lab Chip 2015, 15, 766. 25431832 10.1039/c4lc01013b

[206] L.‐H. Hung , A. P. Lee , J. Med. Biol. Eng. 2007, 27, 1.

[207] S. O. Hotaling , UVM Honors College Senior Theses, University of Vermont 54, 2015.

[208] Y. Tabata , Tissue Eng. 2003, 9, 5. 10.1089/107632703322495574 14633377

[209] K. Oda , Y. Matsuoka , A. Funahashi , H. Kitano , Mol. Syst. Biol. 2005, 1, 1. 10.1038/msb4100014 PMC1681468 16729045

[210] M. Rodrigues , L. G. Griffith , A. Wells , Stem Cell Res. Ther. 2010, 1, 1. 10.1186/scrt32 PMC2983445 20977782

[211] S. Almubarak , H. Nethercott , M. Freeberg , C. Beaudon , A. Jha , W. Jackson , R. Marcucio , T. Miclau , K. Healy , C. Bahney , Bone 2016, 83, 197. 26608518 10.1016/j.bone.2015.11.011 PMC4911893

[212] a) D. J. Prockop , N. Engl. J. Med. 2012, 367, 2353; 23234518 10.1056/NEJMcibr1210178

[213] N. Monteiro , A. Martins , R. L. Reis , N. M. Neves , Regener. Ther. 2015, 1, 109. 10.1016/j.reth.2015.05.004 PMC6581799 31245450

[214] R. L. Siegel , K. D. Miller , A. Jemal , CA: Cancer J. Clin. 2016, 66, 7. 26742998 10.3322/caac.21332

[215] B. Gomes , I. Moreira , S. Rocha , M. Coelho , M. D. C. Pereira , J. Nanopharm. Drug Delivery 2013, 1, 335.

[216] W. Feng , W. Nie , C. L. He , X. J. Zhou , L. Chen , K. X. Qiu , W. Z. Wang , Z. Q. Yin , ACS Appl. Mater. Interfaces 2014, 6, 8447. 24745551 10.1021/am501337s

[217] M. Z. Zhang , C. L. Xu , L. Q. Wen , M. K. Han , B. Xiao , J. Zhou , Y. C. Zhang , Z. Zhang , E. Viennois , D. Merlin , Cancer Res. 2016, 76, 7208. 27742685 10.1158/0008-5472.CAN-16-1681 PMC5161640

[218] J. Wang , P. Mi , G. Lin , Y. X. Wang , G. Liu , X. Chen , Adv. Drug Delivery Rev. 2016, 104, 44. 10.1016/j.addr.2016.01.008 PMC5226392 26805788

[219] H. Y. Yoon , H. R. Kim , G. Saravanakumar , R. Heo , S. Y. Chae , W. Um , K. Kim , I. C. Kwon , J. Y. Lee , D. S. Lee , J. C. Park , J. H. Park , J. Controlled Release 2013, 172, 653. 10.1016/j.jconrel.2013.09.008 24055507

[220] A. Mokhtarzadeh , A. Alibakhshi , H. Yaghoobi , M. Hashemi , M. Hejazi , M. Ramezani , Expert Opin. Biol. Ther. 2016, 16, 771. 26998622 10.1517/14712598.2016.1169269

[221] A. Tamura , N. Yui , J. Biol. Chem. 2015, 290, 9442. 25713067 10.1074/jbc.M115.636803 PMC4392250

[222] A. Tamura , N. Yui , Polym. J. 2017, 49, 527.

[223] A. K. A. Silva , D. Letourneur , C. Chauvierre , Theranostics 2014, 4, 579. 24723980 10.7150/thno.7688 PMC3982129

[224] G. Y. Lee , J.‐H. Kim , K. Y. Choi , H. Y. Yoon , K. Kim , I. C. Kwon , K. Choi , B.‐H. Lee , J. H. Park , I.‐S. Kim , Biomaterials 2015, 53, 341. 25890732 10.1016/j.biomaterials.2015.02.089

[225] L. S. Liu , H. L. He , M. Y. Zhang , S. S. Zhang , W. L. Zhang , J. P. Liu , Biomaterials 2014, 35, 8002.24947229

[226] S. Danhauser‐Riedl , E. Hausmann , H. D. Schick , R. Bender , H. Dietzfelbinger , J. Rastetter , A. R. Hanauske , Invest. New Drugs 1993, 11, 187. 7505268 10.1007/BF00874153

[227] R. Satchi‐Fainaro , R. Duncan , C. M. Barnes , in Polymer Therapeutics II: Polymers as Drugs, Conjugates and Gene Delivery Systems, Vol. 193, (Eds.: Satchi‐Fainaro R., Duncan R.), Springer, New York 2006, p. 1.

[228] R. Duncan , Nat. Rev. Cancer 2006, 6, 688. 16900224 10.1038/nrc1958

[229] E. Kumazawa , Y. Ochi , Cancer Sci. 2004, 95, 168. 14965368 10.1111/j.1349-7006.2004.tb03199.x PMC11158227

[230] K. Inoue , E. Kumazawa , H. Kuga , H. Susaki , N. Masubuchi , T. Kajimura , Adv. Exp. Med. Biol. 2003, 519, 145. 12675213 10.1007/0-306-47932-X_9

[231] O. Soepenberg , M. J. de Jonge , A. Sparreboom , P. de Bruin , F. A. Eskens , G. de Heus , J. Wanders , P. Cheverton , M. P. Ducharme , J. Verweij , Clin. Cancer Res. 2005, 11, 703. 15701859

[232] a) M. Harada , J. Imai , S. Okuno , T. Suzuki , J. Controlled Release 2000, 69, 389; 10.1016/s0168-3659(00)00320-5 11102679

[233] P. F. Bassi , A. Volpe , D. D'Agostino , G. Palermo , D. Renier , S. Franchini , A. Rosato , M. Racioppi , J. Urol. 2011, 185, 445. 21167517 10.1016/j.juro.2010.09.073

[234] R. J. Sylvester , W. Oosterlinck , J. A. Witjes , Eur. Urol. 2008, 53, 709. 18207317 10.1016/j.eururo.2008.01.015 PMC2587437

[235] A. Gabizon , D. Papahadjopoulos , Biochim. Biophys. Acta 1992, 1103, 94. 1309663 10.1016/0005-2736(92)90061-p

[236] J. K. Kim , K. H. Han , J. T. Lee , Y. H. Paik , S. H. Ahn , J. D. Lee , K. S. Lee , C. Y. Chon , Y. M. Moon , Clin. Cancer Res. 2006, 12, 543. 16428498 10.1158/1078-0432.CCR-05-1730

[237] M. E. Davis , Mol. Pharmaceutics 2009, 6, 659. 10.1021/mp900015y 19267452

[238] W. Liang , J. K. W. Lam , in Molecular Regulation of Endocytosis (Ed: Ceresa D. B.), InTech, Croatia, European Union 2012.

[239] J. E. Zuckerman , M. E. Davis , Nat. Rev. Drug Discovery 2015, 14, 843. 26567702 10.1038/nrd4685

[240] a) P. L. Tan , Regener. Med. 2010, 5, 181;

[241] a) Bellerophon BCM LLC , 2010;

[242] a) M. F. Pritchard , L. C. Powell , G. E. Menzies , P. D. Lewis , K. Hawkins , C. Wright , I. Doull , T. R. Walsh , E. Onsoyen , A. Dessen , R. Myrvold , P. D. Rye , A. H. Myrset , H. N. Stevens , L. A. Hodges , G. MacGregor , J. B. Neilly , K. E. Hill , D. W. Thomas , Mol. Pharmaceutics 2016, 13, 863; 10.1021/acs.molpharmaceut.5b00794 26833139

[243] M. C. Roco , J. Nanopart. Res. 2011, 13, 1335.

[244] P. Satalkar , B. S. Elger , P. Hunziker , D. Shaw , Nanomedicine 2016, 12, 893. 26772431 10.1016/j.nano.2015.12.376

[245] a) D. Dheer , D. Arora , S. Jaglan , R. K. Rawal , R. Shankar , J. Drug Targeting 2016, 1, 1; 10.3109/1061186X.2016.1172589 27030377

[246] S. Wilhelm , A. J. Tavares , Q. Dai , S. Ohta , J. Audet , H. F. Dvorak , W. C. W. Chan , Nat. Rev. Mater. 2016, 1, 16014.

[247] E. Szymańska , K. Winnicka , Mar. Drugs 2015, 13, 1819. 25837983 10.3390/md13041819 PMC4413189

[248] B. H. Morrow , G. F. Payne , J. Shen , J. Am. Chem. Soc. 2015, 137, 13024. 26383701 10.1021/jacs.5b07761 PMC4697947

[249] U. Prabhakar , H. Maeda , R. K. Jain , E. M. Sevick‐Muraca , W. Zamboni , O. C. Farokhzad , S. T. Barry , A. Gabizon , P. Grodzinski , D. C. Blakey , Cancer Res. 2013, 73, 2412. 23423979 10.1158/0008-5472.CAN-12-4561 PMC3916009

[250] a) A. Gabizon , M. Bradbury , U. Prabhakar , W. Zamboni , S. Libutti , P. Grodzinski , Lancet 2014, 384, 2175; 25625382 10.1016/S0140-6736(14)61457-4 PMC6615547

[251] C. H. Heldin , K. Rubin , K. Pietras , A. Ostman , Nat. Rev. Cancer 2004, 4, 806. 15510161 10.1038/nrc1456

[252] P. Ruenraroengsak , J. M. Cook , A. T. Florence , J. Controlled Release 2010, 141, 265. 10.1016/j.jconrel.2009.10.032 19895862

[253] S. Goel , D. G. Duda , L. Xu , L. L. Munn , Y. Boucher , D. Fukumura , R. K. Jain , Physiol. Rev. 2011, 91, 1071. 21742796 10.1152/physrev.00038.2010 PMC3258432

[254] A. Richmond , Y. Su , Dis. Models Mech. 2008, 1, 78. 10.1242/dmm.000976 PMC2562196 19048064

[255] H. M. Braakhuis , S. K. Kloet , S. Kezic , F. Kuper , M. V. Park , S. Bellmann , M. van der Zande , S. Le Gac , P. Krystek , R. J. Peters , I. M. Rietjens , H. Bouwmeester , Arch. Toxicol. 2015, 89, 1469. 25975987 10.1007/s00204-015-1518-5 PMC4551544

[256] F. De Cicco , P. Russo , E. Reverchon , C. A. García‐González , R. P. Aquino , P. Del Gaudio , Carbohydr. Polym. 2016, 147, 482. 27178955 10.1016/j.carbpol.2016.04.031

[257] M. Greenwood‐Goodwin , E. S. Teasley , S. C. Heilshorn , Biomater. Sci. 2014, 2, 1627. 25309741 10.1039/C4BM00142G PMC4188404

[258] D. Kuraitis , Z. Arzhangi , A. Hyatt , B. Vulesevica , K. Merrett , J. Zhang , Open Tissue Eng. Regener. Med. J. 2012, 5, 4.

[259] J. P. McQuilling , J. Arenas‐Herrera , C. Childers , R. A. Pareta , O. Khanna , B. Jiang , E. M. Brey , A. C. Farney , E. C. Opara , Transplant. Proc. 2011, 43, 3262. 22099771 10.1016/j.transproceed.2011.10.030 PMC3241996

[260] C. M. Mierisch , S. B. Cohen , L. C. Jordan , P. G. Robertson , G. Balian , D. R. Diduch , Arthroscopy: J. Arthroscopic Relat. Surg. 2002, 18, 892. 10.1053/jars.2002.36117 12368788

[261] W. Li , T. Guan , X. Zhang , Z. Wang , M. Wang , W. Zhong , H. Feng , M. Xing , J. Kong , ACS Appl. Mater. Interfaces 2015, 7, 3018. 25347385 10.1021/am504456t

[262] A. Mohandas , B. S. Anisha , K. P. Chennazhi , R. Jayakumar , Colloids Surf., B 2015, 127, 105. 10.1016/j.colsurfb.2015.01.024 25660093

[263] Y. Parajó , I. d'Angelo , A. Welle , M. Garcia‐Fuentes , M. J. Alonso , Drug Delivery 2010, 17, 596. 20883178 10.3109/10717544.2010.509357

[264] G. H. Choi , H. J. Lee , S. C. Lee , Macromol. Biosci. 2014, 14, 496. 24227631 10.1002/mabi.201300368

[265] L. Bian , D. Y. Zhai , E. Tous , R. Rai , R. L. Mauck , J. A. Burdick , Biomaterials 2011, 32, 6425. 21652067 10.1016/j.biomaterials.2011.05.033 PMC3134110

[266] H. Lu , L. Lv , Y. Dai , G. Wu , H. Zhao , F. Zhang , PLoS One 2013, 8, e69950. 23894564 10.1371/journal.pone.0069950 PMC3720934

[267] F.‐M. Chen , Z.‐W. Ma , G.‐Y. Dong , Z.‐F. Wu , Acta Pharmacol. Sin. 2009, 30, 485. 19305420 10.1038/aps.2009.15 PMC4002268

[268] S. Suarez , G. N. Grover , R. L. Braden , K. L. Christman , A. Almutairi , Biomacromolecules 2013, 14, 3927. 24053580 10.1021/bm401050j PMC3910395

[269] J. Xie , H. Wang , Y. Wang , F. Ren , W. Yi , K. Zhao , Z. Li , Q. Zhao , Z. Liu , H. Wu , C. Gu , D. Yi , Cardiovasc. Ther. 2013, 31, e12. 22954162 10.1111/j.1755-5922.2012.00317.x

[270] L. Zhao , K. Zhang , W. Bu , X. Xu , H. Jin , B. Chang , B. Wang , Y. Sun , B. Yang , C. Zheng , H. Sun , RSC Adv. 2016, 6, 34081.

[271] K. Y. Choi , H. Chung , K. H. Min , H. Y. Yoon , K. Kim , J. H. Park , I. C. Kwon , S. Y. Jeong , Biomaterials 2010, 31, 106. 19783037 10.1016/j.biomaterials.2009.09.030

[272] Z. Xu , Y. Wang , L. Zhang , L. Huang , ACS Nano 2014, 8, 3636. 24580381 10.1021/nn500216y PMC4004320

[273] E. Salva , L. Kabasakal , F. Eren , N. Ozkan , F. Cakalagaoglu , J. Akbuga , Nucleic Acid Ther. 2012, 22, 40. 22217324 10.1089/nat.2011.0312

[274] Q. Zhao , B. Han , Z. Wang , C. Gao , C. Peng , J. Shen , Nanomedicine 2007, 3, 63. 17379170 10.1016/j.nano.2006.11.007

[275] C. Zhang , W. Wang , T. Liu , Y. Wu , H. Guo , P. Wang , Q. Tian , Y. Wang , Z. Yuan , Biomaterials 2012, 33, 2187. 22169820 10.1016/j.biomaterials.2011.11.045

[276] H. Guo , Q. Lai , W. Wang , Y. Wu , C. Zhang , Y. Liu , Z. Yuan , Int. J. Pharm. 2013, 451, 1. 23618965 10.1016/j.ijpharm.2013.04.025

[277] D. G. Ahn , J. Lee , S. Y. Park , Y. J. Kwark , K. Y. Lee , ACS Appl. Mater. Interfaces 2014, 6, 22069. 25487046 10.1021/am505444c

[278] Y. Wang , J. Zhou , L. Qiu , X. Wang , L. Chen , T. Liu , W. Di , Biomaterials 2014, 35, 4297. 24565522 10.1016/j.biomaterials.2014.01.035

[279] S. Cafaggi , E. Russo , R. Stefani , R. Leardi , G. Caviglioli , B. Parodi , G. Bignardi , D. De Totero , C. Aiello , M. Viale , J. Controlled Release 2007, 121, 110. 10.1016/j.jconrel.2007.05.037 17601625

[280] Y. I. Jeong , S. T. Kim , S. G. Jin , H. H. Ryu , Y. H. Jin , T. Y. Jung , I. Y. Kim , S. Jung , J. Pharm. Sci. 2008, 97, 1268. 17674407 10.1002/jps.21103

[281] S. Bisht , A. Maitra , Wiley Interdiscip. Rev.: Nanomed. Nanobiotechnol. 2009, 1, 415. 20049807 10.1002/wnan.43

[282] S. Ganesh , A. K. Iyer , F. Gattacceca , D. V. Morrissey , M. M. Amiji , J. Controlled Release 2013, 172, 699. 10.1016/j.jconrel.2013.10.016 PMC3858515 24161254

[283] a) S. A. Veltkamp , E. O. Witteveen , A. Capriati , A. Crea , F. Animati , M. Voogel‐Fuchs , I. J. van den Heuvel , J. H. Beijnen , E. E. Voest , J. H. Schellens , Clin. Cancer Res. 2008, 14, 7535; 19010872 10.1158/1078-0432.CCR-08-0438

[284] C. Pinnix , G. H. Perkins , E. A. Strom , W. Tereffe , W. Woodward , J. L. Oh , L. Arriaga , M. F. Munsell , P. Kelly , K. E. Hoffman , B. D. Smith , T. A. Buchholz , T. K. Yu , Int. J. Radiat. Oncol., Biol., Phys. 2012, 83, 1089. 22172912 10.1016/j.ijrobp.2011.09.021 PMC3935608

[285] Safety Study of CALAA‐01 to Treat Solid Tumor Cancers. Calando Pharmaceuticals, https://clinicaltrials.gov/ct2/show/NCT00689065?term=CALAA-01&rank=1 (accessed: August 2017).

[286] a) CRLX101 in Combination With Bevacizumab for Recurrent Ovarian/Tubal/Peritoneal Cancer . https://clinicaltrials.gov/ct2/show/study/NCT01652079?term=CRLX101&recr=Recruiting&rank=3 (accessed: August 2017);

[287] Neoadjuvant Chemoradiotherapy With CRLX‐101 and Capecitabine for Rectal Cancer , https://clinicaltrials.gov/ct2/show/study/NCT02010567?term=CRLX101&recr=Recruiting&rank=4 (accessed: August 2017).

[288] a) Alternative Dosing for CRLX101 Alone and With Avastin in Advanced Solid Tumors, 2015 , https://clinicaltrials.gov/ct2/show/study/NCT02648711?term=CRLX101&recr=Recruiting&rank=2 (accessed: August 2017);

[289] Trial of CRLX101, a Nanoparticle Camptothecin With Olaparib in People With Relapsed/Refractory Small Cell Lung Cancer, https://clinicaltrials.gov/ct2/show/study/NCT02769962?term=CRLX101&recr=Recruiting&rank=1 (accessed: August 2017).

